# Insight into biodiversity of the recently rearranged genus *Dickeya*

**DOI:** 10.3389/fpls.2023.1168480

**Published:** 2023-06-20

**Authors:** Nicole Hugouvieux-Cotte-Pattat, Jacques Pédron, Frédérique Van Gijsegem

**Affiliations:** ^1^ Microbiologie Adaptation et Pathogénie, UMR 5240 CNRS, University Lyon, INSA Lyon, Villeurbanne, France; ^2^ Institute of Ecology and Environmental Sciences, Sorbonne University, CNRS, INRAE, Paris, France

**Keywords:** *Pectobacteriaceae*, soft-rot, *Musicola*, *Dickeya*, phytopathogens, pectinases

## Abstract

The genus *Dickeya* includes plant pathogenic bacteria attacking a wide range of crops and ornamentals as well as a few environmental isolates from water. Defined on the basis of six species in 2005, this genus now includes 12 recognized species. Despite the description of several new species in recent years, the diversity of the genus *Dickeya* is not yet fully explored. Many strains have been analyzed for species causing diseases on economically important crops, such as for the potato pathogens *D. dianthicola* and *D. solani*. In contrast, only a few strains have been characterized for species of environmental origin or isolated from plants in understudied countries. To gain insights in the *Dickeya* diversity, recent extensive analyzes were performed on environmental isolates and poorly characterized strains from old collections. Phylogenetic and phenotypic analyzes led to the reclassification of *D. paradisiaca* (containing strains from tropical or subtropical regions) in the new genus, *Musicola*, the identification of three water species *D. aquatica*, *D. lacustris* and *D. undicola*, the description of a new species *D. poaceaphila* including Australian strains isolated from grasses, and the characterization of the new species *D. oryzae* and *D. parazeae*, resulting from the subdivision of the species *D. zeae*. Traits distinguishing each new species were identified from genomic and phenotypic comparisons. The high heterogeneity observed in some species, notably for *D. zeae*, indicates that additional species still need to be defined. The objective of this study was to clarify the present taxonomy of the genus *Dickeya* and to reassign the correct species to several *Dickeya* strains isolated before the current classification.

## Introduction

Members of the genus *Dickeya* belong to the family *Pectobacteriaceae* in the order *Enterobacterales* (Adeolu et al., 2016). Most of them are phytopathogenic bacteria and several species have a broad host range; they infect numerous vegetable crops and ornamental plants, including both monocot and dicot plants (Charkowski et al., 2012; Toth et al., 2021; van der Wolf et al., 2021). These pathogens cause either soft rots or vascular wilts in their plant hosts in temperate, tropical and subtropical climates (Charkowski et al., 2012). The soft rot symptoms are due to the action of bacterial pectinases associated with other plant cell wall-degrading enzymes (PCWDEs), which degrade the main structural components of the middle lamella and primary plant cell wall (Van Gijsegem et al., 2021). These devastating plant pathogens have a significant impact on agriculture, causing crop losses both in fields and during storage. *Dickeya* was classified among the top ten most important bacterial plant pathogens based on its economic and scientific impact (Mansfield et al., 2012). While most *Dickeya* strains have been isolated from diseased plants, some strains have also been found in surface water (Parkinson et al., 2014; Potrykus et al., 2016; Hugouvieux-Cotte-Pattat et al., 2019; Oulghazi et al., 2019; Pedron and Van Gijsegem, 2019; Ben Moussa et al., 2022).

The history of *Dickeya* classification began in 2005 with the proposal to place strains formerly designated *Pectobacterium chrysanthemi* (formerly *Erwinia chrysanthemi*) or *Brenneria paradisiaca* into the new genus *Dickeya* (Samson et al., 2005). At this time, the genus *Dickeya* comprised six recognized species, namely *Dickeya chrysanthemi, Dickeya dadantii*, *Dickeya dianthicola, Dickeya dieffenbachiae*, *Dickeya paradisiaca*, and *Dickeya zeae* (Samson et al., 2005). Thereafter, new changes were proposed in the genus *Dickeya*. Members of the species *D. dieffenbachiae* were reclassified as a subspecies of *D. dadantii* (i.e. *D. dadantii* subsp. *dieffenbachiae*) (Brady et al., 2012). Then, classification largely evolved with the identification of new *Dickeya* species and the contribution of genomics. *Dickeya solani* was identified as the causal agent of severe disease outbreaks of potatoes in Europe (van der Wolf et al., 2014). The novel species *Dickeya fangzhongdai* was isolated from pear trees in China (Tian et al., 2016) and from orchids in different countries (Alic et al., 2018). Recently, some members of the heterogeneous species *D. zeae* were reclassified into *Dickeya oryzae* including rice strains (Wang et al., 2020) and *Dickeya parazeae* (Hugouvieux-Cotte-Pattat and Van Gijsegem, 2021). Three new *Dickeya* species were also isolated from water sources. *Dickeya aquatica* was isolated from freshwaters in Scotland and Finland (Parkinson et al., 2014). *Dickeya lacustris* was found in lake water and in the rhizosphere of waterside plants in France (Hugouvieux-Cotte-Pattat et al., 2019). *Dickeya undicola* was found in water samples collected in Malaysia and France (Oulghazi et al., 2019). In addition, old strains from collection isolated in Australia from sugarcane or other *Poaceae* were classified as *D. poaceiphila* (Hugouvieux-Cotte-Pattat et al., 2020). More recently, the species *D. paradisiaca* has been reassigned to the new genus *Musicola*, including two species *Musicola paradisiaca* and *Musicola keenii* (Hugouvieux-Cotte-Pattat et al., 2021). Thus, the genus *Dickeya* currently comprises twelve validly accepted species: *D. aquatica, D. chrysanthemi, D. dadantii, D. dianthicola, D. fangzhongdai, D. lacustris*, *D. oryzae*, *D. paradisiaca, D. poaceiphila, D. solani, D. undicola*, and *D. zeae*.

Assigning the correct bacterial species to new and old *Dickeya* strains is necessary. The large host-range and non-specific symptoms of the soft-rot diseases increase the importance of taxonomy for accurate identification of the causal pathogens (Toth et al., 2021). Phenotypic analyses, mainly based on biochemical and nutritional traits, were previously used to differentiate species. This method remains interesting for a first classification of a large number of strains. However, the taxonomy has rapidly evolved with the use of genetic and genomic tools. Sequencing of the housekeeping genes *recA* or *gapA* is routinely used to refine interspecific phylogenetic positions of strains from the genus *Dickeya* (Parkinson et al., 2009; Suharjo et al., 2014; Cigna et al., 2017). Whole-genome sequence data are now widely used to understand the evolutionary and taxonomic relationships in bacteria. Thus, both phenotypic analysis and comparative genomics are key tools to improve the taxonomy of cultivable strains.

The virulence equipment of the phytopathogenic *Dickeya* species is based on the production and secretion of a battery of PCWDEs (Van Gijsegem et al., 2021). These bacteria have a high capacity of production and secretion of enzymes possessing pectinase, cellulase or protease activity. The maceration symptom is mainly due to the activity of pectate lyases of the PL1 family (Hugouvieux-Cotte-Pattat et al., 2014), acting together with several accessory pectinases, the cellulase CelZ and a few metalloproteases (Van Gijsegem et al., 2021). The multiplication of PCWDEs in *D. dadantii* 3937 is partly due to gene duplications leading to clusters of homologous genes, namely *pelADE*, *pelBC*, *pehXVW*, and *prtABC* (Hugouvieux-Cotte-Pattat et al, 1996; Hugouvieux-Cotte-Pattat et al, 2014). However, virulence is multifactorial and additional virulence determinants have been identified (Van Gijsegem et al., 2021). Different protein secretion systems (T1SS to T6SS) are present in *Dickeya*. The T2SS Out drives the secretion of extracellular pectinases and of the cellulase CelZ; it is essential for virulence and present in all *Dickeya* members. Most *Dickeya* members possess the T1SS Prt devoted to the secretion of the proteases and the T3SS Hrp secreting a few effectors. In contrast, the equipment in T4SS, T5SS or T6SS largely varies among *Dickeya* species (Van Gijsegem et al., 2021). *Dickeya* members produce an array of secondary metabolites involved in plant-bacteria interactions, competition to other microorganisms, or adaptation to diverse environments. In particular, they carry large gene clusters that encode complex non-ribosomal peptide synthetases (NRPS) and polyketide synthases (PKS) (Van Gijsegem et al., 2021).

Many *Dickeya* strains have been stored in international bacterial collections but this material deserves to be better valued (Broders et al., 2022). For example, the analysis of old *Dickeya* strains from the French Collection of Phytopathogenic Bacteria, CFBP (https://cirm-cfbp.fr/) contributed to clarify the taxonomy and epidemiology of the *Pectobacterium* species (Portier et al., 2020) and allowed the description of the two species *D. poaceiphila* and *M. keenii* (Hugouvieux-Cotte-Pattat et al., 2020; Hugouvieux-Cotte-Pattat et al., 2021). Most *Dickeya* strains isolated and analyzed before 2010s came from diseased crop plants, and there was a need to expand exploration to other environments. *Dickeya* strains are more difficult to isolate from environmental samples which contain many other bacteria, whereas rotten tissues contain almost only the causal agent. This approach was however possible thanks to a powerful semi-selective medium (Hélias et al., 2012) which allowed the isolation of the new aquatic species *D. aquatica*, *D. lacustris*, and *D. undicola* (Parkinson et al., 2014; Hugouvieux-Cotte-Pattat et al., 2019; Oulghazi et al., 2019).

The objective of this study was to present an updated view of the taxonomy of the genus *Dickeya* and to reassign the correct species to *Dickeya* strains isolated before the current classification. We resumed knowledge on the genus *Dickeya* and added new data from phenotypic, genomic and phylogenetic analyses. We tried to favour inter-species comparison by applying the analyses uniformly to all the *Dickeya* species. As the taxonomy within the *Dickeya* genus has been subject to several changes over the past 10 years, this update on the genomic and phenotypic biodiversity within this taxon is supported by information on the strains belonging to each *Dickeya* species.

## Materials and methods

### Strains

Strains of each species are listed in **Tables 1**–**7**; **S1**, with the plant hosts, country of origin and isolation date when these data were available. *Musicola* strains are listed in **Table 1**, *D. aquatica*, *D. lacustris*, *D. poaceiphila* and *D. undicola* strains in **Table 2**, *D. dadantii* strains in **Tables 3**; **S1**, *D. dianthicola* and *D. solani* strains in **Tables 4**; **S1**, *D. fangzhongdai* strains in **Tables 5**; **S1**, *D. chrysanthemi* in **Table 6**, and*D. oryzae, D. parazeae* and *D. zeae strains* in **Tables 7**; **S1**.

**Table 1:** The characterized *Musicola* strains.

Strain designations	Origin: Country, year, plant	Identification criteria
*Musicola paradisiaca*		
Species authority: Hugouvieux-Cotte-Pattat et al., 2021		
CFBP4178^T^ (NCPPB2511^T^)	Colombia 1970, *Musa paradisiaca*	genome, phenotype
Ech703	Australia, *Solanum tuberosum*	genome
CFBP3696 (NCPPB4430)	Cuba 1987, *Musa paradisiaca*	genome, phenotype
CFBP3699	Cuba 1987, *Zea mays*	genome, phenotype
CFBP1445	Colombia 1972, *Musa paradisiaca*	genes *gapA*, *recA, dnaX, leuS*
CFBP1446	Colombia 1972, *Musa paradisiaca*	genes *gapA*, *recA, dnaX, leuS*
CFBP1451	Colombia 1972, *Musa paradisiaca*	genes *gapA*, *recA, dnaX, leuS*
CFBP3477	Colombia 1968, *Musa paradisiaca*	genes *gapA*, *recA, dnaX, leuS*
CFBP2811 (NCPPB2512)	Colombia 1973, *Musa paradisiaca*	genes *recA, dnaX, leuS*
CFBP 4179 (NCPPB2513)	Colombia 1973, *Musa paradisiaca*	genes *recA, dnaX, leuS*
NCPPB2477	Jamaica 1972, *Musa paradisiaca*	phenotype
NCPPB2915	Panama (<1977), *Musa* sp.	phenotype
NCPPB2924	Panama (<1977), *Musa* sp.	phenotype
E353	PRC (China)	gene RNA 16S
572 (= Dickey 141)	*Musa paradisiaca*	gene RNA 16S
*Musicola keenii*		
Species authority: Hugouvieux-Cotte-Pattat et al., 2021		
A3967^T^ (CFBP722,		
CFBP8732^T^, LMG31880^T^)	France 1965, *Solanum lycopersicon*	genome, phenotype

**Table 2:** The characterized strains of D. aquatica, D. lacustris, D. poaceiphila and D. undicola.

Strain designations	Origin: Country, year, plant/habitat	Identification criteria
*D. aquatica*		
Species authority: Parkinson et al., 2014		
174/2^T^ (LMG 27354^T^, NCPPB 4580^T^)	UK 2012, river water	genome, phenotype
DW0440	Finland 2005, river water	genome
CSL RW240	UK, river water	genome
181/2	UK 2012, river water	genes *gyrB, infB, rpoB*
Dw054	Finland 2005, river water	16S rRNA gene
Dw0431	Finland, river water	16S rRNA gene
Dw0512	Finland 2005, river water	16S rRNA gene
JDA74 (CFBP8722)	France 2018, *Solanum dulcamara* rhizosphere	gene *gapA*, phenotype
Not clearly identified strains		
Ca3A, Ca3B	Ireland 2016, *Daucus carota*	gene *recA*
*D. lacustris*		
Species authority: Hugouvieux-Cotte-Pattat et al., 2019		
S29^T^ (CFBP8647^T^, LMG308990^T^)	France 2017, lake water	genome, phenotype
isb1	Pakistan 2021, *Homo sapiens* stool	genome
S12 (CFBP8648)	France 2017, lake water	gene *gapA*, phenotype
S39 (CFBP8649)	France 2017, *Solanum dulcamara* rhizosphere	gene *gapA*, phenotype
J114 (CFBP8721)	France 2018, lake water	gene *gapA*, phenotype
S15	France 2017, lake water	gene *gapA*, phenotype
S24	France 2017, lake water	gene *gapA*, phenotype
*D. poaceiphila*		
Species authority: Hugouvieux-Cotte-Pattat et al., 2020		
NCPPB569^T^ (CFBP8731^T^)	Australia 1958, *Saccharum officinarum*	genome, phenotype
CFBP2040	Australia 1980, *Megathyrsus maximus*	genome, phenotype
CFBP1537 (SR149)	Australia 1958, *Saccharum officinarum*	gene *gapA*, phenotype
*D. undicola*		
Species authority: Oulghazi et al., 2019		
2B12^T^ (CFBP8650^T^) LMG30903^T^	Malaysia 2014, lake water	genome, phenotype
FVG1-MFV-O17	France, 2017, surface water	genome, phenotype
FVG10-MFV-A16	France, 2016, surface water	genome, phenotype
Ca8	RC (Taiwan), *Daucus carota*	genes *dnaA, dnaJ, dnaX, gyrB, recA*
CFBP7083 (CITA C-29)	Spain 2005, *Allium cepa*	gene *gapA*

**Table 3:** Characterized strains of *D. dadantii*.

Strain designations	Origin: Country, year, plant/habitat	Identification criteria
*D. dadantii*		
Species authority: Brady et al., 2012		
*D. dadantii* subsp. *dadantii*		
DSM18020^T^ (NCPPB898^T^, CFBP1269^T^)	Comores 1960, Pelargonium	genome
3937 (CFBP3855)	France 1977, Saintpaulia	genome
NCPPB3537 (IPO598)	Peru 1985, potato	genome
BI3-1, Housui2-1, BI1-1	Japan 2016, apple tree	genome
Kousui1-1	Japan 2016, japanese pear tree	genome
Yana2-2, Aka1-1	Japan 2016, peach tree	genome
Kunimi-3	Japan 2018, peach tree	genome
CZ1501	PRC (China) 2016, sweet potato	genome
M2-3	PRC 2019, potato	genome
ICMP 9290	Papua New Guinea 2005, sweet potato	genome
CFBP1245	USA 1945, Philodendron	genes *dnaX, leuS, recA*
CFBP3695	Cuba 1987, corn	genes *dnaX, leuS, recA*
CFBP1443 (NCPPB2351)	USA 1969, Syngonium	genes *dnaX, leuS, recA*
CFBP1444	Honduras 1973, Syngonium	genes *dnaX, leuS, recA*
CFBP1449	USA 1971, Aglaonema	genes *dnaX, leuS, recA*
CFBP1613	France 1974, Euphorbia	genes *dnaX, leuS, recA*
CFBP1891	USA, tabaco	genes *dnaX, leuS, recA*
CFBP2014	France 1974, Dieffenbachiae	genes *dnaX, leuS, recA*
CFBP2593	Peru, potato	genes *dnaX, leuS, recA*
CFBP3694 (CFBP5649)	Cuba 1987, tomato	genes *dnaX, leuS, recA*
CFBP3697	Cuba 1987, sweet potato	genes *dnaX, leuS, recA*
CFBP3698	Cuba 1987, banana	genes *dnaX, leuS, recA*
CFBP3780	Italy, carnation	genes *dnaX, leuS, recA*
CFBP4152	Greece 1985, Philodendron	genes *dnaX, leuS, recA*
CFBP4177	Jamaica 1970, banana	genes *dnaX, leuS, recA*
MAFF301767	eggplant	genes *recA, dnaX, gyrB, rpoD*
SUPP877	carrot	genes *recA, dnaX, gyrB, rpoD*
SUPP2162	strawberry	genes *recA, dnaX, gyrB, rpoD*
Dri1	RC (Taiwan), Drimiopsis	genes *dnaA, dnaJ, dnaX, gyrB, recN*
PD598	Netherlands 1885, Kalanchoe	gene *recA*
PD1132	Netherlands 1985, Gymnocalicium	gene *recA*
PD552	Netherlands 1988, Scindapsus pictus	gene *recA*
NCPPB2477	Jamaica 1972, banana	gene *recA*
NCPPB3065	Brazil 1978, potato	gene *recA*
NCPPB3458	Hungary 1986, Dieffenbachia	gene *recA*
PD753	Netherlands 1986, Eryngium alpinum	gene *recA*
PD1713	Netherlands 1990, Euphorbia milii	gene *recA*
PD916	RC (Taiwan) 1987, Packera	gene *recA*
PD168	Netherlands 1979, Gymnocalicium	gene *recA*
NCPPB2958	USA 1977, sweet potato	gene *recA*
NCPPB3476	Papua New Guinea1986, sweet potato	gene *recA*
NCPPB4097	Denmark 2000, Euphorbia	gene *recA*
IPO1260	Germany, 2007, potato	gene *dnaX*
IPO2017	Netherlands, hyacinth	gene *dnaX*
*D. dadantii* subsp. *dieffenbachiae*		
NCPPB2976^T^ (CFBP2051^T^)	USA 1957, Dieffenbachia	genome
CFBP1152	Italy 1962, Dieffenbachia	genes *dnaX, leuS, recA*
CFBP1237	Germany 1959, Dieffenbachia	genes *dnaX, leuS, recA*
CFBP1360	France 1970, Dieffenbachia	genes *dnaX, leuS, recA*
CFBP1870	Ivory Coast 1976, Dieffenbachia	genes *dnaX, leuS, recA*
CFBP2597	Switzerland 1963, Dieffenbachia	genes *dnaX, leuS, recA*
NCPPB2454	UK 1972, Dieffenbachia	gene *recA*
IPO1259	Germany, potato	gene *dnaX*
*D. dadantii* (unspecified subspecies)		
S3-1	RC (Taiwan) 2002, calla lily (arum)	genome
FZ06	Philippines 2018, banana	genome
A622-S1-A17	France 2017, water	genome

As they are too numerous, only a selection of strains is given, the complete list of *D. dadantii* strains and the latin name of the host plants are given en ***Table S1***.

**Table 4:** Characterized strains of *D. dianthicola* and *D. solani*.

Strain designations	Origin: Country, year, plant/habitat	Identification criteria
*D. dianthicola*		
Species authority: Samson et al., 2005		
NCPPB453^T^(CFBP1200^T^)	UK 1956, carnation	genome
NCPPB3534	Netherlands 1987, potato	genome
RNS04.9	France 2004, potato	genome
WV516	USA 2016, potato	genome
SS70	Pakistan 2017, potato	genome
ME23	USA, potato	genome
GBBC2039	Belgium, potato	genome
MIE34	Switzerland 2013, potato	genome
S4.16.03.P2.4	Morocco 2016, potato	genome
67.19	USA 2019, New Guinea Impatiens	genome
CFBP1805	Denmark 1977, Kalanchoe	genome
CFBP1984	France 1972, Dianthus	genome
CFBP2598	Switzerland, 1982, Kalanchoe	genome
CFBP2982	France 1978, Kalanchoe	genome
CFBP3706	Switzerland 1986, chicory	genome
CFBP6548	France 1994, chicory	genome
59W	USA 2016, water	genome
NY1528B	USA 2016, potato	genome
A260-S21-A16	France 2016, water	genome
Dd31	Serbia 2018, potato	genes *acnA, gapA, icdA, mdh*
MAFF302984	Japan, 1992, yacon	genes *recA, dnaX, gyrB, rpoD*
MAFF311149	Japan 1996, Kalanchoe	genes *recA, dnaX, gyrB, rpoD*
SUPP2525	Japan 2006, carnation	genes *recA, dnaX, gyrB, rpoD*
SUPP2565	Japan 2006, potato	genes *recA, dnaX, gyrB, rpoD*
CFBP1150	Italy 1967, carnation	genes *dnaX, leuS, recA*
CFBP1244 (NCPPB1956)	Netherlands 1966, Dahlia	genes *dnaX, leuS, recA*
CFBP1274 (NCPPB429)	UK 1956, carnation	genes *dnaX, leuS, recA*
CFBP1276 (NCPPB1385)	Romania 1962, Dahlia	genes *dnaX, leuS, recA*
CFBP1353	Netherlands 1969, Begonia	genes *dnaX, leuS, recA*
CFBP1358	France, 1970, Dahlia	genes *dnaX, leuS, recA*
CFBP3702	France, 1984, artichoke	genes *dnaX, leuS, recA*
D18-A1	Japan 2018, fleabane	genes *recA, dnaX*
D18-B1	Japan 2018, butterbur	genes *recA, dnaX*
D19-W1	Japan 2019, water	genes *recA, dnaX*
S4.16.03.P2.18	Morocco 2016, potato	gene *gapA*
CH85/54	Switzerland 1985, potato	gene *gapA*
MG717687	Australia 2017, potato	gene *recA*
NCPPB394	USA, 1957, Chrysanthemum	gene *recA*
NCPPB2421 (PD823)	Netherlands, 1969, Begonia	gene *recA*
PD554	Netherlands 1985, Kalanchoe	gene *recA*
PD788	Netherlands 1987, Chicory	gene *recA*
PD1325	Netherlands, 1989, Kalanchoe	gene *recA*
PD1077	Bangladeshs, 1988, potato	gene *recA*
IPO1302	Spain, potato	gene *dnaX*
IPO2096	Finland 2005, potato	gene *dnaX*
*D. solani*		
Species authority: Van der Wolf et al., 2014		
IPO2222^T^(NCPPB4479^T^)	Netherlands 2007, potato	genome
Ds0432-1	Finland 2004, potato	genome
RNS 05.1.2A	France 2005, potato	genome
RNS 08.23.3.1.A (3337)	France 2008, potato	genome
RNS 07.7.3B	France 2007, potato	genome
GBBC 2040	Belgium 2007, potato	genome
MK16	UK (Scotland), water	genome
IPO2019	Netherlands 2009, hyacinth	genome
PPO 9019	Netherlands 2006, muscari	genome
PPO 9134	Netherlands 2008, hyacinth	genome
IFB0099	Poland 2005, potato	genome
IFB0158, IFB0167	Poland 2009, potato	genome
IFB0212	Poland 2010, potato	genome
IFB0221, IFB0223	Germany 2005, potato rhizosphere	genome
IFB0231	Finland 2008, potato	genome
IFB0311	Poland 2011, potato	genome
IFB0487	Poland 2013, potato	genome
IFB0695	Poland 2014, potato	genome
IFB0417, IFB0421	Portugal 2012, potato	genome
CH05026-1	Switzerland 2005, potato	genome
CH07044	Switzerland 2007, potato	genome
CH9635-1	Switzerland 1996, potato	genome
CH9918-774	Switzerland 1999, potato	genome
MIE35	Switzerland 2005, potato	genome
D12, F012	Russia 2010, potato	genome
M21a	France 2014, potato	genome
Am3a	France 2015, potato	genome
MK10	Israel, potato	genome
A623-S20-A17	France 2017, water	genome
CFBP7085	Spain 2002, potato	genes *dnaX, leuS, recA*
CFBP7373	Syria 2004, potato	genes *dnaX, leuS, recA*
CFBP5647	France (Guadeloupe), tomato	gene *gapA*
20711883	UK 2007, potato	gene *recA*
G-115	Israel 2007, potato	gene *recA*
IPO2093	Finland 2005, potato	gene *dnaX*

Only a selection of strains is given, the complete list of D.dianthicola and D. solani strains and the latin name of the host plants are given en **Table S1**.

**Table 5:** Characterized strains of *D. fangzhongdai*.

Strain designations	Origin: Country, year, plant/habitat	Identification criteria
*D. fangzhongdai*		
Species authority: Tian et al., 2016		
DSM101947^T^(CFBP8607^T^, JS5^T^)	PRC (China) 2009, pear tree	genome, phenotype
B16 (CFBP8496)	Slovenia 2010, Phalaenopsis	genome, phenotype
S1	Slovenia 2012, Phalaenopsis	genome, phenotype
MK7	UK (Scotland), water	genome, phenotype
NCPPB3274	St. Lucia 1983, Aglaonema	genome, phenotype
PA1	PRC 2011, Phalaenopsis	genome
Onc5	PRC 2021	genome
M005	Malaysia 2013, waterfall	genome
M074	Malaysia 2013, waterfall	genome
ND14b	Malaysia 2013, waterfall	genome
908C	Canada 2020, market	genome
AP6	USA 2014, onion	genome
643b	USA 2020, Aglaonema	genome
LN1	PRC 2014, pear tree	genome
QZH3	PRC 2014, pear tree	genome
Secpp1600	PRC 2016, radish	genome
Ph1 to Ph29	RC (Taiwan), Phalaenopsis	genes *dnaA, dnaJ, dnaX, gyrB, recN*
CAS9, IAS4 and TAS1	RC, Welsh onion	genes *dnaA, dnaJ, dnaX, gyrB, recN*
SUPP40	Japan 1982, Welsh onion	genes *recA, dnaX, gyrB, rpoD*
SUPP420	Japan 1985, Clivia	genes *recA, dnaX, gyrB, rpoD*
SUPP1034	Japan 1988, Phalaenopsis	genes *recA, dnaX, gyrB, rpoD*
SUPP1152	Japan 1989, Oncidium	genes *recA, dnaX, gyrB, rpoD*
SUPP1164	Japan 1989, Vanda	genes *recA, dnaX, gyrB, rpoD*
SUPP1352	Japan 1990, Dracaena	genes *recA, dnaX, gyrB, rpoD*
SUPP1399	Japan 1990, Cattleya	genes *recA, dnaX, gyrB, rpoD*
SUPP1539	Japan 1992, Iris	genes *recA, dnaX, gyrB, rpoD*
SUPP2451	Japan 2004, Welsh onion	genes *recA, dnaX, gyrB, rpoD*
SUPP2586	Japan 2004, taro	genes *recA, dnaX, gyrB, rpoD*
SUPP2737	Japan 1989, Phalaenopsis	genes *recA, dnaX, gyrB, rpoD*
SUPP2738	Japan 1990, Oncidium	genes *recA, dnaX, gyrB, rpoD*
MAFF311172	Japan 1998, taro	genes *recA, dnaX, gyrB, rpoD*
PD813	Netherlands 1987, Phalaenopsis	gene *recA*
PD1750	Netherlands 1990, Yucca	gene *recA*
NCPPB2915	Panama 1977, banana	gene *recA*
NCPPB2929	Solomon Islands 1977, taro	gene *recA*
NCPPB3211	Sri Lanka 1982, orchid	gene *recA*
NCPPB3306	UK 1984, Polyscias	gene *recA*

Only a selection of strains is given, the complete list of D. fangzhongdai strains and the latin name of the host plants are given en **Table S1**.

**Table 6:** Characterized strains of *D. chrysanthemi*.

Strain designations	Origin: Country, year, plant/habitat	Identification criteria
*D. chrysanthemi*		
Species authority: Samson et al., 2005		
NCPPB402^T^ (CFBP2048^T^)	USA 1956, *Chrysanthemum morifolium*	genome
NCPPB516 (CFBP1270)	Danemark 1957, *Parthenium argentatum*	genome
Ech1591		genome
NCPPB 3533	USA 1985, *Solanum tuberosum*	genome
EC16, ATCC11662	USA 1956, *Chrysanthemum morifolium*	genome
L11	Malaysia 2014, water	genome
ws52	PRC 2017, *Nicotiana tabacum*	genome
A604-S21-A17	France 2017, water	genome
CH91/70-1	Switzerland 1991, *Solanum tuberosum*	gene *gapA*
CH93/38-317-4	Switzerland 1993, *Solanum tuberosum*	gene *gapA*
CH93/40-24-1	Switzerland 1993, *Solanum tuberosum*	gene *gapA*
CH98/10	Netherlands 1998, *Solanum tuberosum*	gene *gapA*
CFBP1236 (NCPPB1861)	USA 1945, *Parthenium argentatum*	genes *dnaA, dnaJ, dnaX, gyrB, recA*
CFBP1242 (NCPPB427)	USA 1957, *Chrysanthemum maximum*	genes *dnaA, dnaJ, dnaX, gyrB, recA*
CFBP1275 (NCPPB1111)	UK 1961, Dianthus caryophylus	genes *dnaA, dnaJ, dnaX, gyrB, recA*
CFBP 1346	Italy 1969, *Chrysanthemum maximum*	genes *dnaA, dnaJ, dnaX, gyrB, recA*
CFBP1347	Italy 1969, *Chrysanthemum maximum*	genes *dnaA, dnaJ, dnaX, gyrB, recA*
CFBP1348	Italy 1969, *Chrysanthemum maximum*	genes *dnaA, dnaJ, dnaX, gyrB, recA*
CFBP1441	USA, *Dianthus caryophylus*	genes *dnaA, dnaJ, dnaX, gyrB, recA*
CFBP3262	France 1981, *Cichorum intybus*	genes *dnaA, dnaJ, dnaX, gyrB, recA*
CFBP3263	France 1982, *Cichorum intybus*	genes *dnaA, dnaJ, dnaX, gyrB, recA*
CFBP3701	France 1981, *Lycopersicon esculantum*	genes *dnaA, dnaJ, dnaX, gyrB, recA*
CFBP3703	France 1986, *Helianthus annuus*	genes *dnaA, dnaJ, dnaX, gyrB, recA*
CFBP3704	France (La Réunion) 1986, *Cynara scolymus*	genes *dnaA, dnaJ, dnaX, gyrB, recA*
CFBP5847	Brazil 1994, *Daucus carota*	genes *dnaA, dnaJ, dnaX, gyrB, recA*
CFBP6689	France 2002, *Cichorum intybus*	genes *dnaA, dnaJ, dnaX, gyrB, recA*
CFBP7086	Spain 2003, *Solanum tuberosum*	genes *dnaA, dnaJ, dnaX, gyrB, recA*
NCPPB1849	USA 1966, *Parthenium argentatum*	gene *recA*
NCPPB2148	UK 1968, *Euphorbia pulcherrima*	gene *recA*
NCPPB2149	UK 1968, *Euphorbia pulcherrima*	gene *recA*
NCPPB2227	UK 1969, *Chrysanthemum morifolium*	gene *recA*
NCPPB2309	Italy, *Chrysanthemum morifolium*	gene *recA*
NCPPB2899	USA 1976, *Daucus carota*	gene *recA*
NCPPB3930	Brazil, *Lycopersicon esculentum*	gene *recA*
SUPP20	Japan 1983, *Chrysanthemum* sp.	gene *recA*
SUPP1844	Japan 1998, *Chrysanthemum* sp.	gene *recA*
MAFF302132	Japan 1989, *Solanum melongena*	gene *recA*
MAFF311043	Japan 1992, *Chrysanthemum* sp.	gene *recA*
MAFF311151	Japan 1990, *Cichorum intibus*	gene *recA*
IFB0284	Poland 2011, water	gene *recA*
IFB0320	Poland 2011, water	gene *recA*
IFB0336	Poland 2011, water	gene *recA*
PD720	*Kalanchoe*	gene *recA*
PD806		gene *recA*
IPO2117		gene *recA*
SD17-1 to SD17-11		gene *recA*

**Table 7:** Characterized strains of *t*he *Dickeya zeae* complex (*D. oryzae, D. parazeae, D. zeae*).

Strain designations	Origin: Country, year, plant/habitat	Identification criteria
*D. oryzae*		
Species authority: Wang et al., 2020		
ZYY5^T^	PRC (China), rice	genome, phenotype
DZ2Q (CFBP8738)	Italy, rice	genome, phenotype
S20 (CFBP8715)	France 2017, water	genome, phenotype
FVG03	France 2017, water	genome, phenotype
NCPPB3531 (CFBP8729)	Australia, potato	genome, phenotype
EC1, EC2, ZJU1202	PRC, rice	genome
CSL RW192	UK, water	genome
BRIP64262	Australia	genome
A003-S1-M15	France 2015, water	genome
A642-S2-A17	France 2017, water	genome
NCPPB2547	India 1969, corn	gene *gapA*, phenotype
CFBP1271	Egypt 1961, corn	gene *gapA*, phenotype
CFBP3707	Israel 1986, water	gene *gapA*, phenotype
CFBP4148	Japan 1978, rice	gene *gapA*, phenotype
CH91/71-2	Switzerland 1991, potato	gene *gapA*
A10-S1-M15 + 6 st	France 2015, water	gene *gapA*
A223-S2-A16 + 3 st	France 2016, water	gene *gapA*
A443-S1-J17 + 9 st	France 2017, water	gene *gapA*
IFB0324, 0330, 0334	Poland 2011, water	gene *recA*
IPO648 + 3 st	Netherlands, potato	gene *recA*
SUPP410	Japan 1985, millet	gene *recA*
SUPP739	Japan 1977, rice	gene *recA*
MAFF106502	Japan 1984, rice	gene *recA*
SUPP3076	Japan 2014, rice	gene *recA*
BC2880	Korea, corn	gene *recA*
KFB414, 415, 417	Serbia 2019	gene *recA*
B1B2	Australia 2017	gene *recA*
DZ15SB01 + 3 st	Thailand 2015, corn	gene *recA*
IMI389157	India, aloe	gene *recA*
SR120	corn	gene *recA*
*D. parazeae*		
Species authority: Hugouvieux-Cotte-Pattat et al., 2021		
S31^T^ (CFBP8716^T^, LMG8719^T^)	France 2017, water	genome, phenotype
Ech586	USA, Philodendron	genome
A586-S18-A17	France 2017, water	genome
CFBP1531	USA 1966, corn	gene *gapA*
CFBP1596 (NCPPB3731)	France 1974, corn	gene *gapA*
NCPPB2540	USA, corn	gene *recA*
SUPP27	Japan 1980, corn	gene *recA*
MAFF311098	Japan 1990, corn	gene *recA*
PD1619		gene *recA*
*D. zeae*		
Species authority: Samson et al., 2005		
NCPPB2538^T^ (CFBP2052^T^)	USA 1970, corn	genome, phenotype
NCPPB3532	Australia, potato	genome, phenotype
MK19	UK, water	genome
MS1	PRC 2009, banana	genome
MS2	PRC 2012, banana	genome
MS2014	PRC 2014, banana	genome
MS2018	PRC 2018, banana	genome
WH1	PRC 2021, rice roots	genome
A661-S21-A17	France 2017, water	genome
A5272	USA (Hawai)	genome
BRIP64263	Australia	genome
CFBP1268 (NCPPB1851)	USA 1966, corn	gene *gapA*
CFBP6466	France (Martinique) 1991, pineapple	gene *gapA*
CFBP7084	Spain 2005, water	gene *gapA*
MS3	PRC 2012, banana	gene *recA*
NCPPB2339 (CFBP4176)	USA, 1970, Chrysanthemum	gene *recA*
NCPPB2340	USA, 1970, *Chrysanthemum*	gene *recA*
NCPPB2347	Italy 1971, corn	gene *recA*
SUPP1158	Japan 1989, *Calanthe*	gene *recA*
IPO651	potato	gene *recA*
SR171	corn	gene *recA*
Potential new classification		
FVG08	France 2017, water	genome, phenotype
CE1	PRC, Canna	genome
JZL7	PRC 2017, Clivia	genome
PL65	USA (Hawai) 2018, taro	genome
A5410	USA (Hawai) 2007, pineapple	genome

Only a selection of strains is given, the complete list of strains and the latin name of the host plants are given en **Table S1**.

### Growth conditions and phenotypic analysis

To test their growth with different carbon sources, strains were inoculated onto M63 minimal medium plates supplemented with a sole carbon source (2 g l^-1^). The enzyme secretion was assessed on plates containing an enzyme substrate (Hugouvieux-Cotte-Pattat et al., 2019). For the detection of pectinase activity, bacteria were grown on M63 agar plates supplemented with 2 g l^-1^ glycerol and 4 g l^-1^ polygalacturonic acid. After incubation for 24 h at 30°C, plates were flooded with a saturated solution of copper acetate. Clear haloes appear around colonies secreting pectate-lyases. Cellulase activity was detected on M63 agar plates supplemented with 2 g l^-1^ glycerol and 10 g l^-1^ carboxymethylcellulose. After incubation for 24 h at 30°C, the plates were overflowed with 10 mg ml^-1^ Congo red solution for 10 min and washed for 5 min with 1 M NaCl. Clear haloes surround colonies secreting cellulases. Protease production was tested on LB plates supplemented with skim milk (12.5 g l^-1^). After incubation at 30°C for 24 to 48 h, clear haloes are visible around colonies secreting proteases.

### PCR, amplicon sequencing and recA phylogenetic analysis

The strains were subjected to PCR with primers specific for the gene *gapA* (gapAF and gapAR, 0.8 kb amplicon) (Cigna et al., 2017). The PCR products were sequenced in both directions with the same set of primers, using a commercial service (Microsynth France, Vaulx-en-Velin France). These *gapA* sequences were used for strain identification using BlastN comparison and construction of phylogenetic trees including the type strains. In addition, several *recA* and 16S rRNA sequences were retrieved from NCBI databases and used to define or verify the species affectation of the strain. Several strains firstly identified as *E. chrysanthemi* or *Dickeya* sp. were assigned to species that were not recognized at the time of their isolation.

The phylogenetic tree based on the *recA* gene was constructed using the pipeline Phylogeny.fr (http://www.phylogeny.fr/phylogeny.cgi); the nucleotide sequences are aligned with MUSCLE (Edgar, 2004), the phylogenetic tree is reconstructed using the maximum likelihood method implemented in the PhyML program (Guindon and Gascuel, 2003) and graphical representation is performed with TreeDyn (Dereeper et al., 2008). The *recA* sequences were retrieved from the NCBI database; accession numbers are given in the tree.

### Comparative genomic analysis

The dDDH values were calculated using the webserver Genome-to-Genome Distance Calculator (GGDC) version 2.1 with the formula 2 (Meier-Kolthoff et al., 2013). The Average Nucleotide Identity (ANI) based on the Nucleotide MUMmer algorithm (Delcher et al., 2002) was calculated using the JSpecies Web Server with default parameters (Richter and Rosselló-Móra, 2009). The dDDH value of 70% and the cut-off ANI values of 95–96% between two strains were considered for species delineation (Goris et al., 2007).

To determine the phylogenetic position of *Dickeya* species, the phylogenomic tree was constructed from concatenated protein sequences of 963 unique homologous proteins (293566 sites). It was computed using the BioNJ distance method (Criscuolo and Gascuel, 2008). Two hundred bootstrap replicates were performed to assess the statistical support of each node.

## Results and discussion

### The new genus *Musicola*, analysis of old strains from collections

After analysis of a large panel of strains, Samson et al. (2005) proposed the species *D. paradisiaca* which comprised the former *Brenneria paradisiaca* type strain CFBP 4178^T^ and six *E. chrysanthemi* strains (CFBP 1445, CFBP 1446, CFBP 1451, CFBP 3477, CFBP 3696, CFBP 3699). This classification was mainly based on phylogenetic analyses of 16S rRNA gene sequences which suggested that these strains were phylogenetically distant to all *Brenneria* species. Recently, genomic analyses have suggested that the differences between *D. paradisiaca* and other *Dickeya* species justify the creation of a separate genus (Pritchard et al, 2016; Pedron and Van Gijsegem, 2019). The genus *Musicola* was recognized in 2021, on the basis of phenotypic, phylogenetic and genomic arguments (Hugouvieux-Cotte-Pattat et al., 2021). Most *D. paradisiaca* strains were isolated from banana trees (*Musa paradisiaca*) in tropical or subtropical countries (Dickey and Victoria, 1980) (**Table 1**).

The species previously named *D. paradisiaca* was considered as the earliest branching lineage in the *Dickeya* genus in previous evolution studies (Duprey et al., 2019). However, data on the genetic diversity of this species was scarce, and it was poorly characterized at the genomic level. Only two genome sequences were available, including those of the type strain CFBP 4178^T^ (Pritchard et al., 2013). Calculation of aligned fraction values between *Dickeya* and *Musicola* genomes showed that less than 33% of the genomes of these two genera could be aligned together (Hugouvieux-Cotte-Pattat et al., 2021).

A new *Musicola* strain was identified among poorly characterized strains of the CFBP collection. This strain, A3967 (CFBP 722), is related to *M. paradisiaca* CFBP 4178^T^ but showed atypical phenotypes for sugar assimilation. Genomic comparison (ANI and dDDH values of 96.21% and 68.3%, respectively) confirmed the divergence and justified the description of a novel species *Musicola keenii* (Hugouvieux-Cotte-Pattat et al., 2021). Since the main phenotypic differences between *M. keenii* and *M. paradisiaca* members concern sugar assimilation, a simple distinction between the two species can be obtained by testing the bacterial growth in the presence of myo-inositol, melibiose or raffinose as the sole carbon source (**Table 8**). Sequencing of the genomes of two other *Musicola* strains, CFBP 3477 and CFBP 3699, confirms their assignation to the species *M. paradisiaca*, as suggested by phenotypic analysis (**Table 1**).

*Musicola* strains are less equipped than *Dickeya* members in PCWDEs and other virulence factors. They lack genes encoding PelA, PelI, PnlH, PehN, Rhi, PemB, GanA and Prt (**Table 9**). The reduced number of PCWDE genes could explain the low pectinase activity and the absence of protease secretion observed for *Musicola* members (**Table 8**). While the number of T1SS, T3SS, T4SS and T6SS varies among *Dickeya* species, *Musicola* members have none of them. In addition, they are deprived of factors important to deal with the plant stress responses occuring during infection, such as the nitric oxide dioxygenase HmpX that detoxify nitric oxide produced by plants, the *suf* cluster involved in the repair of damaged Fe/S clusters, and the *ind* cluster encoding the ROS scavenging pigment indigoïdine. These dissimilarities suggest notable differences in the virulence strategies of *Dickeya* and *Musicola* members. Indeed, virulence tests showed that the *Musicola* strains have a weak maceration activity on potato tubers and chicory leaves, two dicot models classically used to evaluate the virulence of *Dickeya* strains (Hugouvieux-Cotte-Pattat et al., 2021). With a low pectate lyase activity, *Musicola* strains are less equipped than *Dickeya* strains to degrade the high pectin content of dicot cell wall. *Musicola* strains may be better adapted to monocots such as *Poaceae* whose primary cell wall has a low pectin content (Jarvis et al., 1988).

**Table 8:** Phenotypic comparison of the different *Dickeya* species.

		Carbon sources												Enzyme secretion			Temp.
Species	Strain	Dara	Mel	Mtl	Xyl	Clb	Ino	G6P	GluA	GalA	Dpsi	Ltart	PGA	Pel	Cel	Prt	39°C
*D. dadantii* ssp. *dad.*	3937	w	+	+	+	+	+	+	+	_	_	_	+	+	w	w	+
*D. dadantii* ssp. *dief.*	CFBP 2051^T^	w	_	+	+	+	+	+	+	_	_	_	+	+	+	+	+
*D. solani*	IPO2222^T^	w	+	+	+	w	+	+	+	+	_	_	+	+	+	+	+
*D. dianthicola*	CFBP 1200^T^	_	+	+	+	+	+	+	+	+	_	_	+	+	+	w	_
*D. fangzhongdai*	DSM101947^T^	w	+	+	+	+	+	+	+	_	w	w	+	+	+	+	+
*D. undicola*	2B12^T^	w	+	+	+	ND	+	+	+	+	_	+	+	+	+	+	+
*D. poaceiphila*	NCPPB 569^T^	w	+	+	+	_	_	_	_	_	+	_	_	w	_	w	+
*D. oryzae*	DZ2Q	w	+	+	+	+	+	+	w	_	w	_	+	+	+	+	+
*D. parazeae*	S31^T^	w	+	+	+	_	+	+	w	_	_	w	+	+	+	w	+
*D. zeae*	NCPPB 2538^T^	w	+	+	+	w	+	+	w	_	_	w	+	+	+	+	+
*D. chrysanthemi*	NCPPB 402^T^	_	+	+	+	w	+	+	+		_	_	+	+	+	+	+
*D. aquatica*	174/2^T^	_	+	_	_	_	+	ND	+	_	_	+	+	+	+	w	+
*D. lacustris*	S29^T^	_	+	_	+	_	+	ND	+	_	_	+	+	+	+	+	+
*M. paradisiaca*	NCPPB 2511^T^	w	+	_	_	_	_	_	+	_	_	_	+	w	+	_	+
*M. keenii*	CFBP 8732^T^	w	_	_	+	_	+	+	+	_	+	_	+	w	+	_	+

For carbon source assimilation, the sign - indicates no growth at 72h; +, indicates growth at 24 h; w, indicates weak growth (visible after 48 or 72 h). Dara, D-arabinose; Mel, D-melibiose; Mtl, mannitol; Xyl, D-xylose; Clb, cellobiose; Ino, myo-inositol; G6P, glucose-6-phosphate; GalA, galactonic acid; GluA, gluconic acid; Dpsi, D-psicose; Ltart, L-tartaric acid; PGA, polygalacturonate (pectin backbone).

For PCWDE secretion: +, positive; -, negative (at 24 h). Pel, pectinase; Cel, cellulase; Prt, protease. +, positive; w, weak; -, negative. Growth at the temperature of 39°C was observed after 48 h in LB medium. ND, nt determined.

Only 16 *Musicola* strains have been characterized up to now (**Table 1**). These strains were mainly isolated from banana and in a few cases from another monocot, corn, and from dicots of the *Solanaceae* family, potato and tomato. They were isolated between 1968 and 1987 (**Table 1**). Unfortunately, no *Musicola* strain has been isolated more recently.

### The water species *D. aquatica* and *D. lacustris*, true *Dickeya* or members of another genus?

**Figure 1:**
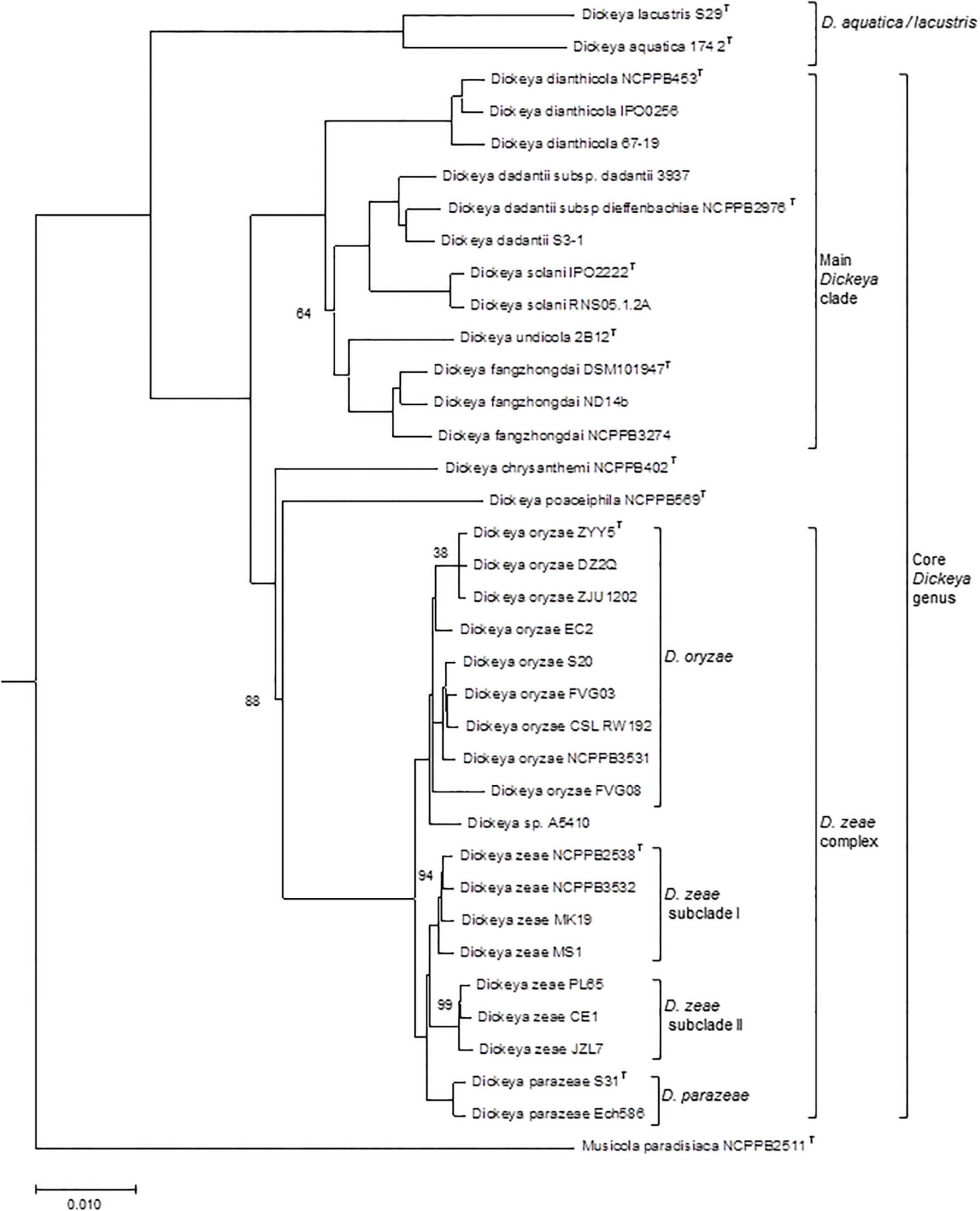
Phylogenomic tree of the *Dickeya* species. The phylogenetic tree, constructed from concatenated amino acid sequences of 963 unique homologous proteins (293566 sites), was computed using the BioNJ distance method. Two hundred bootstrap replicates were performed to assess the statistical support of each node. The tree includes 35 *Dickeya* genomes, representative of the different species and subclades discussed in the text. A *Musicola* genome was used as outgroup. Only boostrap values below 100 are indicated. T, type strain. The scale bar represents the average number of changes per nucleotide position.

Some water isolates belong to phylogenetic groups differing from plant-related *Dickeya* species. The two species *D. aquatica* and *D. lacustris* form a clade distinct from the other *Dickeya* species in phylogenomic studies (Duprey et al., 2019; Hugouvieux-Cotte-Pattat et al., 2019) (**Figure 1**). Some *D. lacustris* strains were found associated to the rhizosphere of bittersweet nightshade (*Solanum dulcamara)*, a weed whose roots are in contact with water (Hugouvieux-Cotte-Pattat et al., 2019). Since water strains deserve less interest than pathogenic isolates, they were less studied. Only a few genomes have been sequenced, three for *D. aquatica* and one for *D. lacustris* (**Table 2**). Very recently, a new *D. lacustris* genome sequence (GCA_027571425.1) became available, it corresponds to strain isb1 isolated in Pakistan from Human stool. The surprising origin of this strain needs further investigation.

**Figure 2:**
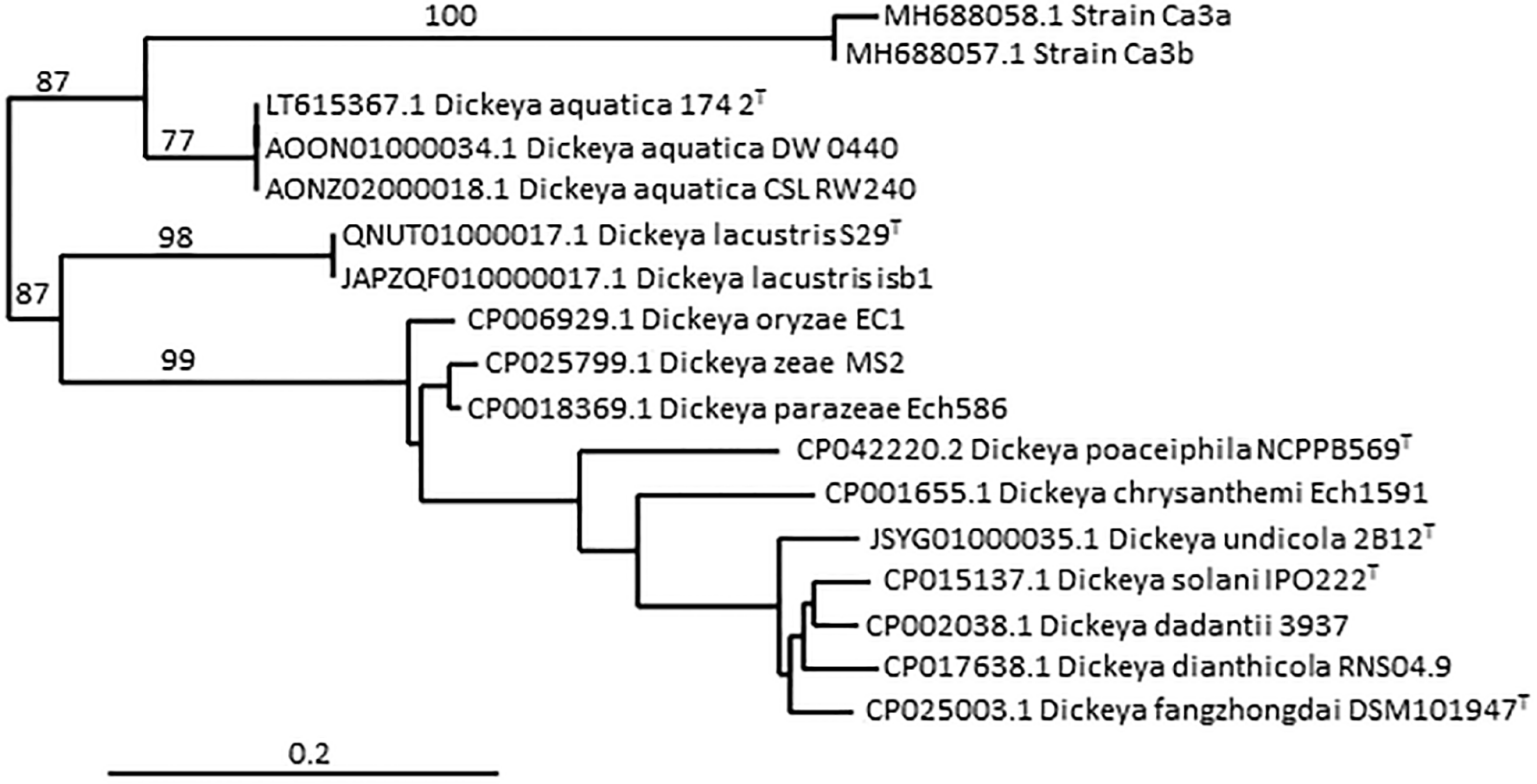
Phylogenetic position of strain Ca3a among the Dickeya species, based on partial recA gene sequences. The tree was constructed using the pipeline Phylogeny.fr (http://www.phylogeny.fr/phylogeny.cgi). The nucleotide sequences are aligned with MUSCLE, the phylogenetic tree is reconstructed using the maximum likelihood method implemented in the PhyML program and graphical representation is performed with TreeDyn. Numbers correspond to bootstrap values (500 replicates). The scale bar represents the average number of substitutions per site. The two strains Ca3a and Ca3b were isolated from carrots in Northern Ireland (Zaczek-Moczydłowska et al., 2019) and their partial recA gene sequences (MH688058 and MH688057, respectively) consisted of 503 nt.

Although there is no formal definition of genus delineation on the basis of genome similarity, recent approaches used the genome aligned fraction to discriminate at genus level, showing that genus boundary corresponds to aligned fraction values around 60% (Barco et al., 2020). Indeed, for strains of the ten species *D. chrysanthemi, D. dadantii, D. dianthicola, D. fangzhongdai, D. oryzae*, *D. parazeae*, *D. poaceiphila, D. solani, D. undicola* and *D. zeae*, paired aligned fractions correspond to at least 58% of the genome lengths (Hugouvieux-Cotte-Pattat et al., 2021). These ten species constitute a coherent group corresponding to the “core *Dickeya* genus*”* (**Figure 1**). With aligned fractions of 73% between D*. lacustris* and *D. aquatica* genomes, these two species correspond to a homogeneous group. In contrast, the aligned fraction of *D. lacustris* and *D. aquatica* genomes is less than 41% with other *Dickeya* species (Hugouvieux-Cotte-Pattat et al., 2021). At the phenotypic level, D*. lacustris* and *D. aquatica* showed some metabolic particularities in comparison to the ten species of the “core *Dickeya* genus”, such as the non-assimilation of xylose or mannitol (**Table 8**). However, their PCWDE equipment is quite similar to that of other *Dickeya* species. They lack a few accessory pectinases PnlG, PehN and PemB (**Table 9**), but they have the capacity to macerate different plants in laboratory conditions (Duprey et al., 2019). A notable difference is their ecology as *D. lacustris* and *D. aquatica* seem to inhabit only water. An exception could be an isolate from carrot in Northern Ireland which was putatively identified as *D. aquatica* on the basis of a partial *recA* sequence (Zaczek-Moczydłowska et al., 2019). However, phylogenetic analysis of the corresponding sequences (MH688057, MH688058) suggests that strain Ca3 is quite distant from *D. aquatica* (**Figure 2**). Further analysis will be required to clarify the classification of this strain. The isolation of new strains and sequencing of new genomes is essential in order to obtain more information on the phenotypic and genetic diversity of these water species.

**Table 9:** Repartition of the main PCWDEs in the different *Dickeya* species.

			*Dickeya* sp.												*Musicola* sp.	
Enzymatic activity	CAZY family	Gene name	Ddad	Dsol	Ddian	Dfang	Dund	Dpoa	Dor	Dpara	Dzeae	Dchrys	Daq	Dlac	Mparad	Mkeen
Pectin lyase	PL1	*pnlG*	1	1	1	1	1	0	1	0	1	0	0	0	0	0
		*pnlH*	0	0	1	0	0	0	0	0	0	1	0	1	1	1
Pectate lyase	PL1	*pelA/D/E*	3	3	2	3	3	1	3	3	3	2	2	3	2	2
		*pelB/C*	2	2	2	2	2	1	2	2	2	2	2	2	2	2
		*pelZ*	1	1	1	1	1	0	1	1	1	1	1	1	1	1
	PL2	*pelW*	1	1	1	1	1	1	1	1	1	1	1	1	1	1
	PL3	*pelI*	1	1	1	1	1	1	1	1	1	1	1	1	0	0
	PL9	*pelL*	1	1	1	1	1	0	1	1	1	1	1	1	1	1
		*pelN*	1	0	1	1	1	1	1	1	1	1	1	1	1	1
		*pelX*	1	1	1	1	1	1	1	1	1	1	1	1	1	1
	PL10	*pel10*	0	0	0	1	0	0	0	0	0	0	1	0	0	0
Rhamnogalacturonate	PL4	*rhiE*	1	1	1	1	1	0	0	0	1	1	0	1	0	0
lyase	PL26	*rhiF*	1	1	1	1	0	0	0	0	0	1	0	1	0	0
Polygalacturonase	GH28	*pehV/W/X*	3	3	2	1	1	1	1	1	1	2	1	1	1	1
		*pehK*	1	0	1	1	1	0	1	1	1	0	1	1	1	1
		*pehN*	1	1	1	1	1	0	0	0	1	1	0	0	0	0
Pectin methylesterase	CE8	*pemA*	1	1	1	1	1	1	1	1	1	1	1	1	1	1
		*pemB*	1	1	1	1	1	0	0	0	0	1	0	0	0	0
Pectin acetylesterase	CE10	*paeY*	1	1	1	1	1	0	1	1	1	1	1	1	1	1
	CE12	*paeX*	1	1	1	1	1	1	1	1	1	1	1	1	1	1
Ferruloyl esterase	CE10	*faeD*	1	1	1	1	1	1	1	1	1	1	1	1	1	1
		*faeT*	1	1	1	1	1	0	1	1	1	1	1	1	1	1
Cellulase	GH5	*celZ*	1	1	1	1	1	0	1	1	1	1	1	1	1	1
Galactanase	GH 53	*ganA*	1	1	1	1	1	0	1	1	1	1	1	1	0	0
Protease		*prtA/B/C*	3	3	2	3	3	1	3	3	3	3	3	3	0	0
		*prtG*	1	1	0	1	0	0	1	1	1	1	1	1	0	0

Data are given for the type strain of each species. The families of enzymes that degrade or modify polysaccharides are described in the CAZy database (http://www.cazy.org/) (Drula et al., 2022). GH, glycoside hydrolase; PL, polysaccharide lyase; CE, carbohydrate esterase.

### Analysis of old strains from collections, the species *Dickeya poaceiphila*

The phenotypic and genetic analysis of poorly characterized strains from collections allowed the characterization of the species *Dickeya poaceiphila*, including a group of strains isolated in Australia from sugarcane and other *Poaceae* (commonly known as grasses) (Hugouvieux-Cotte-Pattat et al., 2020). Between 1955 and 1957, a plant disease affecting sugarcane in Australia was designated by bacterial mottle (Steindl, 1964). The symptoms consisted of chlorotic striping of the leaves which were heavily invaded by bacteria as were also the stems, causing severe stunting and wilting (Dowson and Hayward, 1960). Symptoms of chlorosis and wilting were also observed on various grasses occurring near sugarcane fields, such as *Pennisetum purpureum* (elephant grass), *Megathyrsus maximus* (Guinea grass), and *Brachiaria mutica* (para grass). The strain NCPPB 569^T^ was sampled during this outbreak in Australia; its genome sequence was reported in 2013 (Pritchard et al., 2013). Genomic comparisons indicated that this strain was a candidate for assignment to a novel *Dickeya* species (Pritchard et al., 2016; Duprey et al., 2019) and the species *D. poaceiphila* was recognized in 2020 (Hugouvieux-Cotte-Pattat et al., 2020). In addition to the type strain, it includes strain CFBP 2040 whose genome was also sequenced (dDDH values of 90.2%) and strain CFBP 1537 also isolated in Australia (**Table 2**).

The three *D. poaceiphila* strains showed substantial phenotypic differences compared to other *Dickeya* species. They produce a low level of pectinases and proteases and no cellulase activity (**Table 8**). This low secretion of PCWDEs is associated with a low capacity to macerate potato tubers and chicory leaves (Hugouvieux-Cotte-Pattat et al., 2020). The absence of extracellular cellulase activity in *D. poaceiphila* results from the absence of the cellulase gene *celZ* present in all other *Dickeya* species (**Table 9**). The weak protease production could be due to the presence of a single *prt* gene in *D. poaceiphila*, while several *prt* genes are present in other *Dickeya* species (**Table 9**). Similarly, the low level of pectinase activity could be due to a low number of pectinase genes. *D. poaceiphila* has only two genes encoding secreted pectate lyases of the polysaccharide lyase family 1 (PL1), one of the *pelADE* cluster and one of the *pelBC* cluster. PL1 are responsible for the major pectate lyase activity of *Dickeya* (Hugouvieux-Cotte-Pattat et al., 2014) and the other *Dickeya* species contain 5 to 6 genes encoding PL1 pectate lyases (**Table 9**). *D. poaceiphila* is the only *Dickeya* species lacking the pectate lyase genes *pelL* and *pelZ*. It also lacks the accessory pectinase genes *pnlG, pnlH, pehK, pehN, pemB* and the galactanase gene *ganA*. It has only one polygalacturonase gene of the cluster *pehXVW* (**Table 9**). Several gene duplications leading to clusters of *pel, peh* or *prt* genes in other *Dickeya* species are not found in *D. poaceiphila.* This restricted number of PCWDE activities and genes could explain why *D. poaceiphila* strains are poorly effective in causing soft rot symptoms (**Tables 8**, **9**) and instead produce other types of symptoms, such as stunting or wilting (Dowson and Hayward, 1960).

Additional phenotypic differences between *D. poaceiphila* and other *Dickeya* species were observed for sugar assimilation, including an absence of growth with D-gluconic acid, D-fructose-6-phosphate, D-glucose-1-phosphate, D-glucose-6-phosphate, *myo*-inositol, or polygacturonate (PGA), the pectin backbone (**Table 8**). The absence of growth of *D. poaceiphila* on pectin or polygalacturonate may result from the inactivation of *kduI*, a gene involved in the intracellular pectin catabolic pathway (Hugouvieux-Cotte-Pattat et al., 1996). This gene is annotated as a pseudogene in *D. poaceiphila* because it has an internal deletion of 555 bp. A defect in pectin assimilation is clearly a factor that could affect the bacterial growth in macerated plant tissues. It is less troublesome for a species that does not cause maceration of its host plants but other types of symptoms.

While *D. poaceiphila* strains show clear differences in phenotypic and genomic features from strains of the other characterized *Dickeya* species, phylogenomic studies confirmed that these strains clearly belong to the genus *Dickeya* (Hugouvieux-Cotte-Pattat et al., 2020). However, the *D. poaceiphila* genomes appear to be the smallest ones (4.22-4.32 Mb) among the *Dickeya* species and they have the lowest GC content (52.60-52.70%) (**Table 10**). A reduced genome size is consistent with the numerous gene losses observed in this species and suggests an evolution of these genomes towards an adaptation to a more restricted habitat. This could correspond to an adaptation to a specialized host range and type of symptom as *D. poaceiphila* has only been shown to cause wilt disease on monocot herbaceous plants of the *Poaceae* family.

**Table 10:** Range of genome size and GC% for the *Dickeya* and *Musicola* species.

Species	Genome number	Genome size (Mb)	GC%	Intraspecies ANI	Intraspecies dDDH
*D. dadantii*	17	4.66-5.35	55.90-56.50	>96.3	>68.9
*D. solani*	44	4.81-5.07	56.10-56.38	>98.7	>89.2
*D. dianthicola*	76	4.68-4.91	55.60-56.00	>97.3	>76.4
*D. fangzhongdai*	16	4.93-5.18	56.44-56.90	>96.1	>68.5
*D. undicola*	3	4.35-4.61	54.50	>98.9	>92.9
*D. poaceiphila*	2	4.02-4.32	52.60-52.80	98.8	90.3
*D. oryzae*	10	4.53-4.75	53.30-53.70	>96.0	>67.9
*D. parazeae*	3	4.71-4.82	53.60-53.70	>98.6	>88.1
*D. zeae*	21	4.56-4.93	53.30-53.70	>96.1	>68.3
*D. chrysanthemi*	8	4.62-4.81	54.19-54.51	>96.2	>68.9
*D. aquatica*	3	4.34-4.50	53.35-53.60	>99.9	>99.1
*D. lacustris*	2	4.30-4.31	53.10	100	100
*M. paradisiaca*	2	4.63-4.68	55.00	100	99.9
*M. keenii*	1	4.40	54.40	_	_

For each species, are given the number of available genomes, their size range and their GC% range (data from the NCBI genome database, February 2023) and the intraspecies ANI and dDDH values.

Only three *D. poaceiphila* strains have been characterized and only two genome sequences have been reported (**Table 2**). Since the three strains were isolated from the same country, isolation and characterization of new strains from different origins will be necessary to access to the diversity of the species *D. poaceiphila* and to confirm its genomic divergence and potential specialization within the genus *Dickeya*.

### The main *Dickeya* clade includes *D. dadantii, D. dianthicola, D. fangzhongdai, D. solani* and *D. undicola*

Using phylogenetic studies, two clades are observed in the core *Dickeya* genus (**Figure 1**). One includes *D. chrysanthemi*, *D. poaceiphila, D. oryzae, D. parazeae* and *D. zeae*. The other, called the “main *Dickeya* clade”, regroups the five species *D. dadantii*, *D. dianthicola*, *D. fangzhongdai, D. solani* and *D. undicola* (**Figure 1**). These five species form a clade sharing high ANI values (89 to 94%) and share more than 50% of their protein families (around 2600) including most virulence genes (Pedron and Van Gijsegem, 2019). They have a large equipment in PCWDEs (**Table 9**). Their genomes encode at least ten pectate lyases (PelB, PelC, PelD, PelE, PelI, PelL, PelN, PelW, PelX and PelZ), the rhamnogalacturonate lyase RhiE, the predicted pectin lyase PnlG, the pectin acetyl esterases PaeX and PaeY, the feruloyl esterases FaeD and FaeT, and the two pectin methyl esterases PemA and PemB (**Table 9**). The arsenal of polygalacturonases is more variable, ranging from three to five, including at least PehN and PehX. All species of the main *Dickeya* clade produce the cellulase CelZ and the galactanase GanA. Their genome harbor at least two protease genes (**Table 9**). While the siderophore clusters *cbs* and *acs* belongs to the *Dickeya* core genome, most clusters encoding secondary metabolite biosynthesis are members of the *Dickeya* accessory genomes, as they variably distributed among species and sometimes among strains of the same species (**Table 11**).

Despite their high genetic proximity and sharing of virulence related genes, the closeness and host ranges of these five *Dickeya* species differ, ranging from narrow to wide host ranges and genomic diversities. The two species *D. dadantii* and *D. fangzongdai* are even able to cause tree diseases in orchards, such as the bleeding canker necrosis of pear trees (Tian et al., 2016) and the bacterial quick decline of apple trees (Fujikawa et al., 2019).

### *D. dadantii*, a wide host range, the model strain 3937

Noticeably, the *D. dadantii* species includes the strain 3937 that has been used for a long time as a model of the genus *Dickeya* for genetic studies leading to the characterization of virulence factors, including the different enzymes constituting the PCWDE equipment, and the regulatory network controlling expression of the virulence genes (Hugouvieux-Cotte-Pattat et al., 1996; Hugouvieux-Cotte-Pattat et al., 2014; Reverchon et al., 2016). The 3937 genome was the first *Dickeya* genome to be sequenced (Glasner et al., 2011).

Among all *Dickeya* species, *D. dadantii* has the widest host range and geographical distribution. It was isolated in all continents, from several dicots and monocot plants. More than 100 strains have been isolated from at least 25 countries and 26 different plants, including apple, peach and pear trees, vegetables (potato, tomato, eggplant, carrot, sweet potato), and numerous ornamentals (*Aglaonema, Dieffenbachia, Drimiopsis*, *Euphorbia, Kalenchoe, Pelargonium, Phylodendron, Saintpaulia, Syngonium*, etc). It was occasionally found in corn (*Zea mays*), cactus (*Gymnocalicium*), alpine plants (*Erygium alpinum*) and water (**Table 3**; **Table S1**).

First described as a separate species, *D. dieffenbachiae* was included as a subspecies of *D. dadantii* (*D. dadantii* subsp. *dieffenbachiae*) since both species type strains shared 96.6 ANI and 71.4% dDDH values. The main *D. dadantii* clade was renamed *D. dadantii* subsp. *dadantii* (Brady et al., 2012). The main phenotypic difference between the two subspecies is the assimilation of melibiose (**Table 8**). *D. dadantii* subsp. *dieffenbachiae* strains were nearly all isolated from ornamentals of the genus *Dieffenbachia* (*Araceae* family). With the isolation of new Asiatic strains of *D. dadantii* (Fujikawa et al., 2019; Wei et al., 2021; Tan et al., 2022), the heterogeneity within this species may increase. The strain S3-1 isolated from an *Araceae* (*Zantedeschia aethiopica*) in Taiwan (Wei et al., 2021) is distinct from members of both *D. dadantii* subspecies, with 96.3-96.7 ANI values and 68-72% dDDH values. This raises the possibility of an additional *D. dadantii* subspecies including strains S3-1, FZ06 and A622-S1-A17 (**Table 3**, **Figure 1**). A classification at the subspecies level can be justified when each subspecies has a specific feature or is linked to a particular host. However, newly isolated strains are rarely classified at this level, except when there is a clear phenotypic difference between the subspecies. So far, no specific traits have been described for the three *D. dadantii* strains not assigned to a recognized subspecies. Nevertheless, with 17 genome sequences available (**Table 3**), the genomic diversity of the *D. dadantii* species is exemplified by the large range of genome sizes, from 4.66 to 5.35 Mb (**Table 10**).

Another intriguing type of diversity among *D. dadantii* members concerns the *vfm* cluster which is present in all *Dickeya* strains (**Table 11**). The VFM molecule plays a major role in intercellular communication by acting as a quorum sensing molecule (Nasser et al., 2013). However, differences in the enzymatic specificity of the proteins VfmO and VfmP generate the production of different analogs of the VFM molecule, which vary according to the strain (Hugouvieux-Cotte-Pattat et al., 2022). As these analogs are differentially recognized, signaling through the Vfm quorum sensing system is limited to strains belonging to compatible groups. For instance, the *D. dadantii* strains are divided in two groups and the production of two different VFM analogs affects the intra-species communication (**Table 11**).

**Table 11:** Clusters encoding secondary metabolites in the *Dickeya* species.

				*Dickeya* sp.												*Musicola*sp.	
Cluster	Gene nbr	Product	Activity	Ddad	Dsol	Ddian	Dfang	Dund	Dpoa	Dor	Dpara	Dzeae	Dchrys	Daq	Dlac	Mparad	Mkeen
*vfm*	26	VFM molecule	quorum-sensing	**+**	**+**	**+**	**+**	**+**	**+**	**+**	**+**	**+**	**+**	**+**	**+**	**+**	**+**
* *		VFM group (s)	VFM analogs	I, IV	III, IV	I	I, III, IV	III, IV	V	II, III	III, IV	III, IV	II, III, IV	IV	IV	VI	VI
*cbs*	16	chrysobactin	siderophore	**+**	**+**	**+**	**+**	**+**	**+**	**+**	**+**	**+**	**+**	**+**	**+**	**+**	**+**
*acs*	6	achromobactin	siderophore	**+**	**+**	**+**	**+**	**+**	**+**	**+**	**+**	**+**	**+**	**+**	**+**		
*ind*	3	indigoidin	antioxidant	**+**	**+**	**+**	**+**	**+**	**+**	**+**	**+**	**+**	**+**	**+**	_	_	_
*sol/ssm*	11	solanimycin	antifungal	**v+**	**+**	**_**	**+**	**v+**	**+**	**v+**	**+**	**+**	**_**	**+**	**_**	**_**	**_**
*ooc*	20	oocydin A	antifungal	**_**	**+**	**+**	**v-**	**_**	**_**	**v**	**_**	**_**	**v**	**v-**	**_**	**+**	**_**
*zms*	17	zeamine	phytotoxin, antibiotic	**_**	**+**	**_**	**+**	**_**	**_**	**v-**	**_**	**_**	**_**	**_**	**_**	**_**	**_**
*car*	8	carbapenem	antibiotic	**v-**	**_**	**_**	**_**	**+**	**_**	**v-**	**_**	**v**	**v+**	**+**	**+**	**_**	**_**
*cfa*	10	coronafacic acid	phytotoxin	**v-**	**_**	**_**	**_**	**_**	**+**	**_**	**_**	**_**	**_**	**_**	**_**	**_**	**_**
cluster A	16	unknown	unknown	**_**	**_**	**_**	**_**	**_**	**_**	**_**	**_**	**_**	**_**	**+**	**_**	**_**	**_**
cluster B	9	unknown	unknown	**_**	**_**	**_**	**_**	**_**	**_**	**_**	**_**	**_**	**_**	**_**	**_**	**+**	**_**
cluster C	37	unknown	unknown	**_**	**_**	**_**	**_**	**_**	**_**	**_**	**_**	**_**	**_**	**_**	**_**	**+**	**_**

Data are given for the sequenced genomes of each species. +, present; - absent; v, variable; v+, variable but mostly present; v- variable but mostly absent.

### *D. dianthicola*, a potato pathogen known since 1950s

The two species *D. dianthicola* and *D. solani* deserved special attention because they caused severe damages in the important crop potato. Most analyzed genomes are from strains isolated from diseased potatoes introducing a severe bias for diversity analyses. Despite its reputation of potato pathogen, *D. dianthicola* has a broad host range (van der Wolf et al., 2021). It was first described in an outbreak on carnation in Europe in the 1950s. Nearly 300 strains have now been characterized, including about 80% potato isolates (**Table 4**; **Table S1**). Other isolates were reported from vegetables, such as chicory or artichoke, and ornamentals, frequently carnation, kalanchoe, begonia and dahlia (**Table 4**). Potato infections in Europe have been detected since the 1970s but losses caused by *D. dianthicola* remained generally low and sporadic (Toth et al., 2011) except in Switzeland and the Netherlands where *D. dianthicola* dominated till the advent of *D. solani* in the 2000s (Pedron et al., 2021; Pedron et al., 2022). Since 2015, *D. dianthicola* caused a severe blackleg outbreak in the US that originated from Maine and further spread in at least eighteen US states (Charkowski, 2018). *D. dianthicola* also caused a blackleg outbreak in Western Australia in 2017 (Wright et al., 2018).

All *D. dianthicola* genomes contain the cluster *ooc* involved in the biosynthesis of an oocydin A-like molecule that may contribute to the maintenance of these species in the potato environment, by favouring the competition with other microorganisms (Brual et al., 2023). At the genomic level, *D. dianthicola* has a truncated pectate lyase gene *pelA* and lacks two protease genes (**Table 9**). At the phenotypic level, it is the sole member of the main *Dickeya* clade unable to grow with D-arabinose as the sole carbon source and it is the sole *Dickeya* species unable to grow at 39°C (**Table 8**). Such phenotypic differences could reflect a divergent adaptation of *D. dianthicola* to environmental conditions.

Due to the large interest for this potato pathogen, more than 70 *D. dianthicola* genome sequences are now available (**Tables 4**, **10**). Most show strong relatedness illustrated by ANI values greater than 99% and clonal origin of the strains isolated during the US potato outbreak (Ge et al., 2021). Analysis of additional genomes highlights the diversity in *D. dianthicola*, with two strains isolated from potato in the Netherlands in 1975 and from impatiens in the US in 2019 (Liu et al, 2021) (**Table 4**), sharing 97 to 98% ANI values with other *D. dianthicola* genomes (Pedron et al., 2022). A recent report of *D. dianthicola* presence in *Asteraceae* weeds (fleabane and butterbur) close to potato fields points to another possible route of transmission from weeds to potato through surface water flow (Aono et al., 2022).

### *D. solani*, a potato pathogen emerging in the 2000s

In contrast to *D. dianthicola, D. solani* has a narrow host range, it was recognized as the agent of a potato outbreak in Europe in the 2000s (Slawiak et al., 2009; van der Wolf et al., 2014), then it spread in Asia (Israel, Syria, Turkey, Georgia, oriental Russia), North Africa (Morocco) and Latin America (Brazil) (van der Wolf et al., 2021). On 65 *D. solani* characterized strains so far (**Table 4**; **Table S1**), only two other hosts have been identified apart to potato, the ornamentals muscari (one strain) and hyacinth (2 strains), and two strains were isolated from water (**Table 4**). A large majority of *D. solani* isolates collected in Europe and the Mediterranean Basin are clonal (Khayi et al., 2015; Golanowska et al., 2018; Blin et al., 2021). Even strains isolated more than 20 years apart are highly related, indicating a high genomic stability (Pedron et al., 2021). Genomic variations in *D. solani* genomes consist mainly in the presence of genes related to phages and even complete prophages (Golanowska et al., 2018; Khayi et al., 2022). The first exception was observed with strain RNS05.1.2A isolated from potato in France, which showed an ANI value of only 98% with other *D. solani* genomes (Khayi et al., 2015). Recently, two other French strains, RNS10-105-1A and A623S-20A-17, isolated from potato and surface water respectively, were shown to belong to the same rare subclade (Khayi et al., 2022). The other genetic variations observed in *D. solani* results from horizontal gene transfers leading to gene replacement. A few potato strains collected in France (RNS07.7.3B, RNS13-30-1A, RNS13-31-1A and RNS13-48-1A) acquired genomic regions related to the RNS05.1.2A subclade (Khayi et al., 2022). Two *D. solani* strains isolated from ornamentals (PPO9019 and PPO9134) acquired gene clusters from *D. dianthicola*. The strain PPO9019 also possesses a 45 kb plasmid identical to a plasmid of *Burholderia ambifaria* (Khayi et al., 2015). Interestingly, *D. solani* and *B. ambifaria* beside sharing an identical plasmid, exhibit similarities in the O-polysaccharide compositions that both contain 6-deoxyaltropyranose (Ossowska et al., 2017). Even if divergences remain rather limited, the *D. solani* strains from ornamentals or water seem to show more diversity than the potato isolates. Strains showing genomic variations were collected in The Netherlands and France and it would be interesting to further analyze the potential *D. solani* diversification in other countries.

A genomic comparison revealed the presence of three genomic regions encoding NRPS, PKS and associated proteins which are present in all *D. solani* genomes but absent in *D. dadantii* 3937 (Garlant et al., 2013; Pedron et al., 2014). First described for some *D. oryzae* strains (Zhou et al., 2011), the cluster *zms* is found in all *D. solani* and *D. fangzhongdai* genomes (**Table 11**). It encodes proteins involved in the biosynthesis of toxins of the zeamine family. The zeamine produced by *D. solani* is involved in antibacterial activity (Effantin et al., 2021; Brual et al., 2023). The *D. solani* cluster *ooc* comprises genes involved in the biosynthesis of an oocydin A-like molecule, a compound inhibiting *Ascomycetes* growth (Effantin et al., 2021; Brual et al., 2023). This cluster is also found in all the *D. dianthicola* genomes and some strains of *D. fangzongdai, D. oryzae, D*. *chryanthemi* and *D. aquatica* (**Table 11**). The cluster *sol* (or *ssm*) produces an antifungal compound recently named solanimycin (Matilla et al., 2022). Solanimycin is active against a broad range of plant-pathogenic fungi and the human pathogen *Candida albicans* (Effantin et al., 2021; Matilla et al., 2022; Brual et al., 2023). The *sol* cluster is conserved in most *Dickeya* species, including all the genomes of *D. fangzongdai*, *D. poaceiphila*, *D. parazeae* and *D. zeae* and in some strains of *D. dadantii, D. undicola* and *D. oryzae* (**Table 11**). The presence of the *sol* cluster appears to be correlated with signs of horizontal genetic transfer (Matilla et al., 2022).

### *D. fangzhongdai*, a tree and orchid pathogen

**Figure 3:**
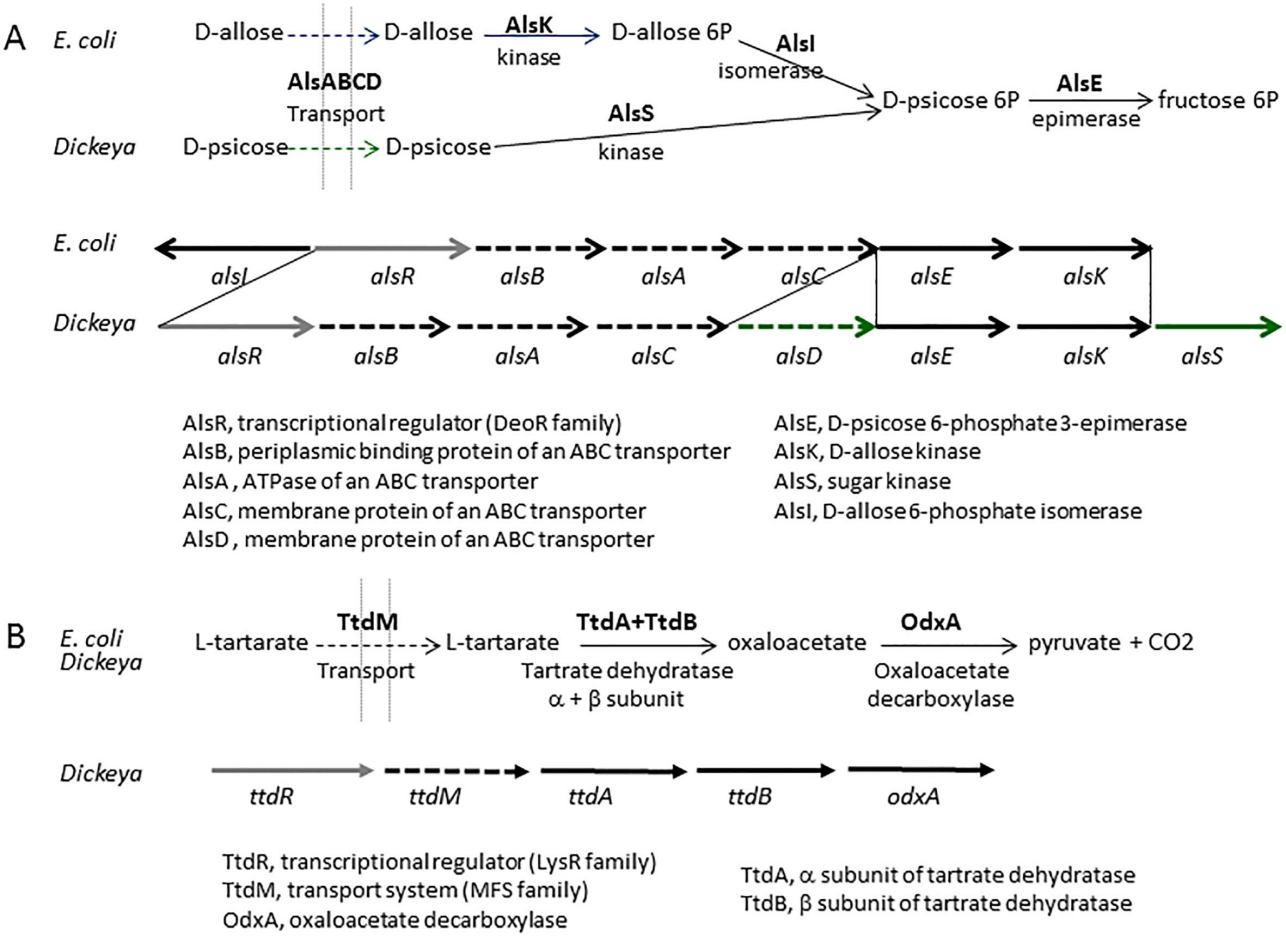
The *Dickeya* clusters involved in D-pscicose and L-tartarate catabolism. **(A)** Comparison of the pathways and gene clusters involved in D-allose and D-pscicose utilization in *E*. *coli* and *Dickeya*, respectively. The *E*. *coli* cluster involved in D-allose utilization contains the additional gene *alsI*, encoding the isomerase necessary for D-allose assimilation. The *Dickeya* cluster involved in D-pscicose utilization includes two additional genes,encoding a second permease (AlsD) and a second sugar kinase (AlsS). The cluster *alsRBACDEKS* is found in the three *Dickeya* species able to utilize D-pscicose, *D. angzhongdai, D. oryzae*, and *D*. *poaceiphila*. **(B)** The L-tartarate catabolic pathway and the *Dickeya* cluster involved in L-tartarate utilization. The three-step pathway of L-tartarate assimilation is biochemically similar in *E*. *coli* and *Dickeya*. The genes *ttdA* and *ttdB* encode the two subunits of tartrate dehydratase. The *Dickeya* genes *ttdR, ttdM* and *odxA* encode a regulator, a transporter and a decarboxylase which belong to different protein families but are predicted to have similar functions. The cluster *ttdRMABodxA* is found in the different *Dickeya* species able to utilize L-tartarate, namely *D*. *aquatica*, *D*. *fangzhongdai, D. parazeae*, *D*. *undicola* and *D*. *zeae*.

*D. fangzhongdai* was the first *Dickeya* species found to infect trees, where it causes bleeding canker necrosis (Tian et al., 2016). Most *D. fangzhongdai* isolates originated from Asia (**Table 5**, **Table S1**), but the species was also identified in Europe, mainly in orchids (Alic et al., 2017). Analysis of old bacterial collections showed that *D. fangzhongdai* was introduced to Europe as early as 1985, through the propagation of ornamental plants (Parkinson et al., 2009; Van Vaerenbergh et al., 2012; Suharjo et al., 2014; Alic et al., 2018). Beside pear trees, *D. fangzhongdai* is able to cause soft rot on orchids (*Phalenopsis, Oncidium, Vanda, Cattleya*), and it is also found in water (Alic et al., 2018) (**Table 5**). Unlike other members of the main *Dickeya* clade, *D. fangzhongdai* strains are able to assimilate the rare sugar D-psicose (**Table 8**). The D-psicose catabolic pathway was not yet characterized but this capacity was also observed for *D. oryzae* and *D. poaceiphila* (**Table 8**). Genome mining in the three *Dickeya* species able to utilize D-psicose demonstrates the correlation between the presence of the gene cluster *alsRBACDEKS* and the bacterial utilization of D-psicose (**Figure 3A**). The importance of this pathways during pathogenesis is unknown but D-psicose was shown to induce some plant defense genes, conferring plant resistance to diseases (Kano et al., 2011).

When analyzed for their maceration ability in laboratory conditions, *D. fangzhongdai* members appear to be more efficient than any other *Dickeya* species (Alic et al., 2018). In addition to classical PCWDE equipment, the *D. fangzhongdai* genome contains a gene encoding a pectate lyase of the rare family PL10 (**Table 9**). PL10 enzymes exhibit catalytic properties similar to those of pectate lyases of the PL1 family such as an alkaline optimal pH, a Ca2+ requirement and a preference for low methylated pectin as substrate (Hugouvieux-Cotte-Pattat et al., 2014). They adopt an (α/α)3 barrel topology in place of the parallel β-helix topology found for PL1 pectate lyases, but the catalytic machinery used by PL10 and PL1 displays a striking resemblance, presumably due to convergent evolution (Charnock et al., 2002).

*D. fangzhongdai* clearly shows intra-species diversity with ANI values ranging from 96 to 99% between its members. The *D. fangzhongdai* strains are distributed in three clades in phylogenetic trees (**Figure 1**). One strain, NCPPB3274, is at the limit of being part of the species, with ANI values of ~96% and dDDH values of 68.6-70.5% with other *D. fangzhongdai* isolates. The *D. fangzhongdai* strain S1 carries a 23 kb plasmid similar to an *Acidovorax* plasmid. Besides genes involved in plasmid replication, stabilization and conjugative transfer, this plasmid contains two streptomycin kinase genes responsible for the resistance to the antibiotic streptomycin (Alic et al., 2019).

*D. fangzhongdai* is particularly well armed in secondary metabolites. In addition to the clusters *zms* and *sol* involved in the biosynthesis of the antibiotic zeamine and the antifungal compound solanimycin, respectively (**Table 11**), all the *D. fangzhongdai* genomes possess additional NRPS/PKS clusters (Alic et al., 2019; Van Gijsegem et al., 2021). Two of them are predicted to be involved in the biosynthesis of cyanobactin-related and thiopeptide-related metabolites, respectively. The atypical strain NCPPB3274 harbours the cluster *ooc* involved in the biosynthesis of an oocydin A-like molecule.

### *D. undicola*, only a water species?

The species *D. undicola* was only recently described after isolation of three strains from surface waters, one from a lake in Malaysia and two from an irrigation canal in the South of France (Oulghazi et al., 2019) (**Table 2**). Phylogenetic analyses identified *D. fangzhongdai* as the closest *Dickeya* species (**Figure 1**). Carbon sources utilization analysis identified galactonic acid as a potential discriminative character for distinguishing between these two species (**Table 8**). The PCWDE repertoire of *D. undicola* is identical to that of *D. dadantii*, except that it produces only one polygalacturonase of the *pelXVW* cluster (**Table 9**). The genes involved in resistance to stresses encountered during plant infection are shared by all species of the main *Dickeya* clade, except that *D. undicola* harbors a truncated flavohemoglobin gene *hmpX* (Van Gijsegem et al., 2021). *D. undicola* genomes contain the *car* locus involved in the synthesis of the β-lactam antibiotic, 1-carbapen-2-em-3-carboxylic acid (carbapenem) as well as genes encoding the carbapenem immunity proteins. This cluster is also found in the water species *D. aquatica* and *D. lacustris*, some strains of *D. chrysanthemi*, *D. oryzae* and *D. zeae* (**Table 11**) and some *Pectobacterium* species (Van Gijsegem et al., 2021).

Despite they were isolated from far regions, the three *D. undicola* strains are closely related as they shared ANI and dDDH values higher than 99 and 92%, respectively, but each genome carries hundreds of specific genes (Pedron and Van Gijsegem, 2019). The low number of genomes sequenced, only three, remains insufficient to relate the closeness of the accessory genomes to the geographical origin. Recently, a new *D. undicola* strain was isolated from carrot in Taiwan (Wei et al., 2021) and we identified as *D. undicola* a strain from the CFBP collection isolated from onion in 2005 in Spain (**Table 2**). These two isolates suggest that *D. undicola* is not only a water species but can also infect plants, perhaps after irrigation with contaminated water. To better understand the *D. undicola* diversity and habitat, it will clearly be interesting to analyze new strains and to sequence the genomes of isolates from diverse origins.

### *D. chrysanthemi*, the type species of the genus *Dickeya*

The species *D. chrysanthemi* is the type species of the genus *Dickeya* (Samson et al., 2005). The type strain was isolated in 1956 from *Chrysanthemum morifolium* displaying signs of soft rot and wilt. Other members of the species have been isolated from various plants, mostly *Chrysanthemum* sp., *Parthenium argentatum*, carnation, sunflower, tobacco, and from several vegetables including potato, tomato, carrot, eggplant, chicory, and artichoke. Several members were also isolated from surface water (**Table 6**).

At the phenotypic level, *D. chrysanthemi* is unable to grow with D-arabinose as the sole carbon source, a feature shared by a few other *Dickeya* species such as *D. dianthicola*, *D. aquatica*, and *D. lacu*stris (**Table 8**). The *D. chrysanthemi* PCWDE equipment is quite typical of the genus *Dickeya*; the genome just lacks the secondary pectinase genes *pnlG* and *pehK*, one *pel* gene of the cluster *pelADE* and the protease gene *prtG* (**Table 9**).

Phylogenomic data suggest that the species *D. chrysanthemi* contains at least two subclades. Since dDDH values of 69%−70% were observed between most distant members, at the border of the species delimitation; this species clearly includes a high genetic diversity (**Table 10**). However, although this species has been known for a long time, only eight *D. chrysanthemi* genome sequences are currently available (**Table 6**); this is insufficient to propose a new classification in this heterogeneous species.

### The *Dickeya zeae* complex: *D. oryzae, D. parazeae, D. zeae* and maybe others

Since classification of the strains previously classified as *D. zeae* has recently evolved, the term “*D. zeae* complex” was used to describe all these isolates (Hugouvieux-Cotte-Pattat and Van Gijsegem, 2021). In addition to the species *D. zeae*, the *D. zeae* complex comprises the species *D. oryzae*, initially described for a rice isolate (Wang et al., 2020), and *D. parazeae* whose type strain is a water isolate (Hugouvieux-Cotte-Pattat and Van Gijsegem, 2021). Several genome sequences of strains belonging to the *D. zeae* complex are avaible in public databases but most of the corresponding strains have not been deposited in collections and are not publicly available for phenotypic analyses, restraining their microbiological characterization.

A high diversity was noticed within genomes of the *D. zeae* complex which form distinct clades in phylogenetic analyses (Pedron and Van Gijsegem, 2019). The two main clades includes a group of genomes clustering with the *D. zeae* type strain, and another group clustering with the *D. oryzae* type strain (**Figure 1**). It should be noted that the majority of strains previously classified as *D. zeae* are reclassified as *D. oryzae* (**Table 7**; **Table S1**). In addition, a separate branch is formed by strains of the species D. *parazeae*. The strain CE1 clusters with the strains PL65 and JZL7 isolated from taro and clivia, respectively (Hu et al., 2018; Boluk et al., 2021) (subclade II, **Figure 1**). These three strains share dDDH values of about 90%, and values of ~68%, ~65% and ~56% with *D. zeae* subclade I (**Figure 1**), *D. parazeae* and *D. oryzae*, respectively. Thus, they may correspond to a novel species of the *D. zeae* complex and, indeed, strain PL65 was recently proposed as the type strain of a new species, *Dickeya colocasiae* (Boluk et al., 2022), but this classification is not officially recognized. The *D. zeae* strain A5410, which was isolated from pineapple (Boluk et al., 2021), also forms a separate branch (**Figure 1**); it shares dDDH similarities of 66−70% to *D. oryzae*, and 57-58% to *D. zeae* and *D. parazeae*. Strain FVG08, isolated from water is another example of strains not clearly positionned in the phylogenetic tree (**Figure 1**); it is at the limit of belonging to the species *D. oryzae* with dDDH values of 67.1-69.2% with other *D. oryzae* strains. These examples highlight the residual heterogeneity of the *D. zeae* complex. Interesting, the different *D. oryzae* subclades show some host preference, suggesting an evolution towards host adaptation. The rice strains form a homogeneous subclade also including a millet strain. A second *D. oryzae* branch brings together mostly maize strains. A third branch includes isolates from water and potato; these later may result from contaminations of potato crops by irrigation water (Hugouvieux-Cotte-Pattat and Van Gijsegem, 2021).

The *D. oryzae*, *D. parazeae* and *D. zeae* strains exhibit high secreted pectinase, protease and cellulase activities (**Table 8**). Their repertoire of PCWDEs is similar to those of the main *Dickeya* clade (**Table 9**). Differences were observed only for accessory pectinases; the genes encoding PnlH, PemB and RhiF are absent in all genomes of the *D. zeae* complex. The genes encoding PehN and RhiE are absent in *D. parazeae* and *D. oryzae*. Some differences in their virulence equipment disclose particularities of rice strains among *D. oryzae* members. The *D. oryzae* rice strains possess the zeamine cluster *zms*, the gene *pnlG*, and the genes *cyt* encoding entomotoxins but not the glycopeptidase gene *avrL.* In contrast, the other *D. oryzae* strains possess *avrL* but not *zms*, *pnlG*, or *cyt*. The toxin zeamine was shown to be involved in the rice foot rot disease caused by *D. oryzae* (Zhou et al., 2011). Despite its name, zeamine is not produced by *D. zeae* but by *D. solani*, *D. fangzhondai* and some *D. oryzae* strains isolated from rice (**Table 11**).

A phenotypic difference was observed between *D. oryzae* and *D. zeae* for the assimilation of L-tartarate, a compound abundant in many fruits (**Table 8**) (Hugouvieux-Cotte-Pattat and Van Gijsegem, 2021). Genomic comparison allowed the identification of the gene cluster *ttdRMAB-odxA* encoding the L-tartarate catabolic pathway in *Dickeya* (**Figure 3B**). This pathway was previously described in *Salmonella typhimurium* and *Escherichia coli* (Hurlbert and Jakoby, 1965), but the *Dickeya* cluster encodes proteins of different families sharing the same function, indicating a convergent evolution among *Enterobacterales*. The six species *D. zeae*, *D. parazeae D. fangzhongdai*, *D. aquatica*, *D. lacustris* and *D. undicola* are able to assimilate L-tartarate (**Table 8**). Their genome contains the cluster *ttdRMAB-odxA*, confirming the correlation between phenotypic and genomic data.

To reassign strains previously classified as *D. zeae*, we took advantage of the many available *recA* sequences and we sequenced the *gapA* gene from available strains (**Table 7**). This study also gives information into the host range or habitat of each species. Most *D. oryzae* isolates come from corn and they are frequently found in potato, rice and water. Most of the *D. zeae* isolates originate from corn, banana, potato, and water. Similarly, the *D. parazeae* strains were mostly isolated from corn and water (**Table 7**). Thus, members of the *D. zeae* complex are noticeably most common on monocots (corn, rice, banana, pineapple), but also frequent in surface water and in a few dicots, such as potato.

## Conclusion

Since the definition of the genus *Dickeya* and the six first historic species *D. chrysanthemi, D. dadantii*, *D. dianthicola, D. dieffenbachiae*, *D*. *paradisiaca*, and *D. zeae* (Samson et al., 2005), mainly based on phenotypes and 16S rRNA comparison, we recently faced numerous assignation modifications and new species occurrence due to sharp genomic comparisons of known *Dickeya* members, new disease outbreaks, data mining in old collections and enlargement of habitat surveys. Genomic analyses resulted in the re-assignation of *D*. *paradisiaca* to the new genus *Musicola*. They also led to the split of *D. zeae* into three species and putatively more, given the high diversity among the *D. zeae* members. The severe outbreak in potato crops in Europe in the 2000s led to the identification of the new species *D. solani*. Similarly, *D. fangzhongdai* was described in 2016 after outbreaks in apple trees and in orchids. The atypical species *D. poaceiphila* was described after analysis of the diversity of old strains present in collections. Surveys of surface waters allowed the characterization of three new species, *D. aquatica*, *D. lacustris* and *D. undicola*, absent or rarely present on plants. New investigations in other environments such as soils but also alternative hosts such as insects or nematodes could also expand the range of *Dickeya* diversity. It should also be noted that, apart from the *Musicola* strains isolated almost exclusively in Latin America, international bacterial collections contain very few isolates from Central and South America or Central Africa. Given their climatic particularities, investigations in these underscored geographical locations should also broaden our vision of the *Dickeya* diversity.

Even if they are usually described as pathogens presenting a large host range, some *Dickeya* species seem to have a preference for herbaceous monocots. For instance, *D. poaceiphila* was only isolated from *Poaceae* and members of *D. oryzae*, *D. parazeae* and *D. zeae* were more often isolated from monocots than from dicots (**Tables 2**, **7**). Similarly, *Musicola* members were mostly identified on monocots (banana trees) (**Table 1**). Pectate lyases are critical virulence factors for causing the soft rot symptoms; as these enzymes have no strict substrate specificity (Hugouvieux-Cotte-Pattat et al, 2014) they allow *Dickeya* to attack a large range of plants. Adaptation to a preferential host may depend on the variety and abundance of PCWDEs. For instance, *D. poaceiphila* has the poorest PCWDE repertoire among *Dickeya* members and shows a low production of pectate lyases that would disfavor the breakdown of the cell walls rich in pectin. *D. poaceiphila* is less well equipped to degrade the high pectin content of dicot cell wall and may be better adapted to monocots whose primary cell wall has low pectin content, such as *Poaceae* (Jarvis et al., 1988). The inactivation of pectin catabolism in *D. poaceiphila* is also a strong argument indicating that pectin degradation has become a secondary virulence factor for this species that produces mostly symptoms of chlorosis and wilting.

Genomic analyses are powerful but not suitable to the first screening of a large number of isolates as they need sequencing facilities and remains costly in time and resources. Thus, the phenotypic analysis of simple growth traits should not be forgotten for a preliminary strain classification (**Table 8**). In addition, the sequencing of a quality marker gene, such as *recA* or *gapA* is recommended in order to obtain a reliable taxonomic assignation of newly isolated strains. In any case, a correct classification of phytopathogenic bacteria in genera and species requires many strains of various origins (in terms of host plants, habitats and countries), a phenotypic analysis of these strains and a sufficient number of sequenced genomes to propose a solid phylogeny.

## Data availability statement

The original contributions presented in the study are included in the article/**Supplementary Material**. Further inquiries can be directed to the corresponding author.

## Author contributions

NH-C-P – conceptualization of the study, phenotypic analysis, writing, reviewing, and editing of the manuscript. JP – comparative genomics, phylogenetic analysis. FV – genome analysis, writing, reviewing, and editing of the manuscript. All authors contributed to the article and approved the submitted version.

## Supplementary material

Supplementary Table 1 Complete list of *D. dadantii, D. dianthicola, D. solani, D. fangzhongdai D. oryzae*, and *D. zeae* strains.

Supplementary Table 2 List of the genome sequences used in this study with their accession numbers and references.
